# A time differentiated dietary intervention effect on the biomarkers of exposure to pyrethroids and neonicotinoids pesticides

**DOI:** 10.1016/j.isci.2022.105847

**Published:** 2022-12-22

**Authors:** Elina Ioannou, Stavros Oikonomou, Nikolaos Efthymiou, Andria Constantinou, Thibaut Delplancke, Pantelis Charisiadis, Konstantinos C. Makris

**Affiliations:** 1 Cyprus International Institute of Environmental and Public Health, Cyprus University of Technology, Limassol, Cyprus; 2 Nutrition & Dietetics Department, Limassol General Hospital, State Health Services Organization, Limassol, Cyprus

**Keywords:** Toxicology, Exposure assessment, Food science

## Abstract

Tailoring medical models to the right person or risk subgroups delivered at the right time is important in personalized medicine/prevention initiatives. The CIRCA-CHEM randomized 2x2 crossover pilot trial investigated whether the consumption of fruits/vegetables within a time-restricted daily window would affect urinary biomarkers of exposure to neonicotinoids (6-chloronicotinic acid, 6-CN) and pyrethroids (3-phenoxybenzoic acid, 3-PBA) pesticides, a biomarker of oxidative damage (4-hydroxynonenal, 4-HNE) and the associated urinary NMR metabolome. A statistically significant difference (p *< 0.001*) in both creatinine-adjusted 6-CN and 3-PBA levels was observed between the two-time dietary intervention windows (morning vs. evening). In the evening intervention period, pesticides biomarker levels were higher compared to the baseline, whereas in the morning period, pesticide levels remained unchanged. Positive associations were observed between pesticides and 4-HNE suggesting a diurnal chrono-window of pesticide toxicity. The discovery of a chronotoxicity window associated with chrono-disrupted metabolism of food contaminants may find use in personalized medicine initiatives.

## Highlights

• Time of day that food is consumed affects the body burden of pesticides
• Higher urinary metabolized pesticides when foods are consumed at night vs morning
• Association of metabolized pesticides with a marker of oxidative damage
• Evidence of chronodisrupted metabolism of ubiquitous pesticides in common foods

## Graphical abstract

**Figure:**
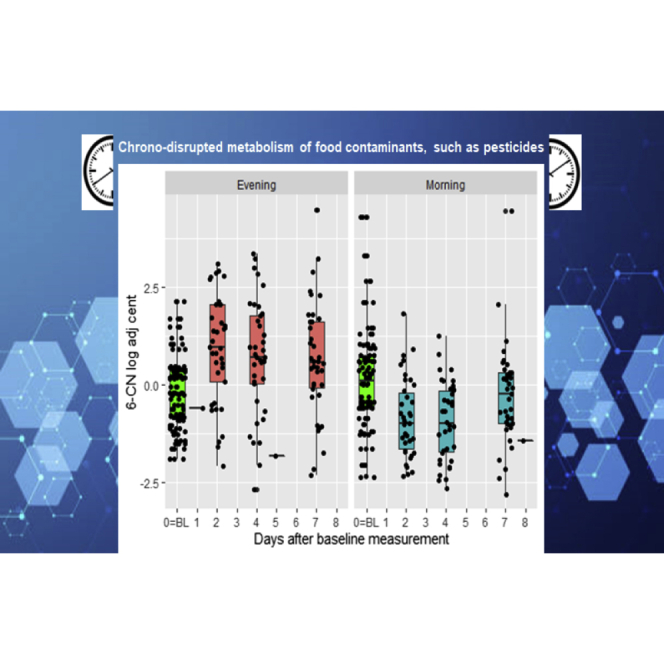

## Introduction

It is well documented that low fruit and vegetable consumption is associated with increased mortality and morbidity risk.^1^^,^^2^^,^^3^ Healthy eating promotion schemes are accompanied by recommendations to achieve daily consumption of at least 3 portions of fruit and 2 portions of vegetables (FAO/WHO, 2004).^4^ However, pesticide residues in food and particularly in fruits and vegetables are ubiquitously found, carrying some health risk for the consumers.^5^^,^^6^^,^^7^ Traditional health promotion programs that call for higher intake of fruits and vegetables may be associated with a dampened positive impact of their antioxidant content on human health, because of the concurrent presence of food contaminants, such as pesticide(s) residues; pesticides are considered pro-oxidants and endocrine disruptors associated with neurodevelopmental, metabolic and carcinogenic effects.^8^^,^^78^

Pyrethroids are widely used as a substitute for organophosphate insecticides and are considered the main alternative choice to neonicotinoids.^75^ Accordingly, pyrethroids use is anticipated to increase especially in areas where neonicotinoid use is restricted due to concerns about pollinators.^76^ Nevertheless, there are more than seven neonicotinoid pesticides that account for >25% of the global pesticide market.^77^ Residues of pyrethroids and neonicotinoids are common food contaminants that are typically metabolized during phase I metabolism in the human liver, generating the well-known metabolites 3-PBA and 6-CN, respectively. Alike drugs, pyrethroids and neonicotinoids may follow a hepatic circadian metabolic activity catalyzed by isoenzymes of the CYP450 family.^9^ The main enzymes that oxidize pesticides are CYP1A2, CYP2C9, CYP2C19, and CYP3A4; all except for CYP2C9 are well-known for their rhythmicity.^10^^,^^11^^,^^12^^,^^13^^,^^14^^,^^15^^,^^16^

Chrono-stamped human behaviors are implicated with differentiated metabolism of associated environmental stressors (e.g., chemicals in diet). Earlier findings of our research team showed that the time of exposure in the day dictated the magnitude of the urinary biomarker of exposure to disinfectants/disinfection by-products that are associated with household cleaning activities.^17^ In parallel, research has shown that eating earlier in the daytime is aligned with the circadian metabolism system, reducing appetite and promoting weight loss.^18^^,^^19^^,^^20^^,^^21^^,^^22^ Time-restricted eating has been positively associated with the risk management of chronic metabolic diseases,^23^ but none has studied the effect of time-restricted dietary components consumption (fruits and vegetables) on the associated pesticides’ metabolism and concomitant food contaminant toxicity to human physiology.

In addition, the role of peripheral clocks (liver) and associated biological systems in shaping the magnitude and extent of pesticide metabolism found as residues in common fruits and vegetables remain poorly understood in real-life human settings. To the best of our knowledge, no other randomized controlled trial (RCT) has studied the time-of-day effect on the body burden of pesticides following adherence to a daily dietary consumption pattern of the same dose of fruits and vegetables. We, therefore, aimed to investigate whether the adherence to the same daily consumption pattern of fruits and vegetables (and thus, about the same pesticide residue load) within two different time-restricted windows (morning vs evening) could have a differential effect on the biomarkers of exposure to pesticides and also on the associated urinary metabolome.

## Results

### Demographics, baseline characteristics, and compliance

**Figure 1:**
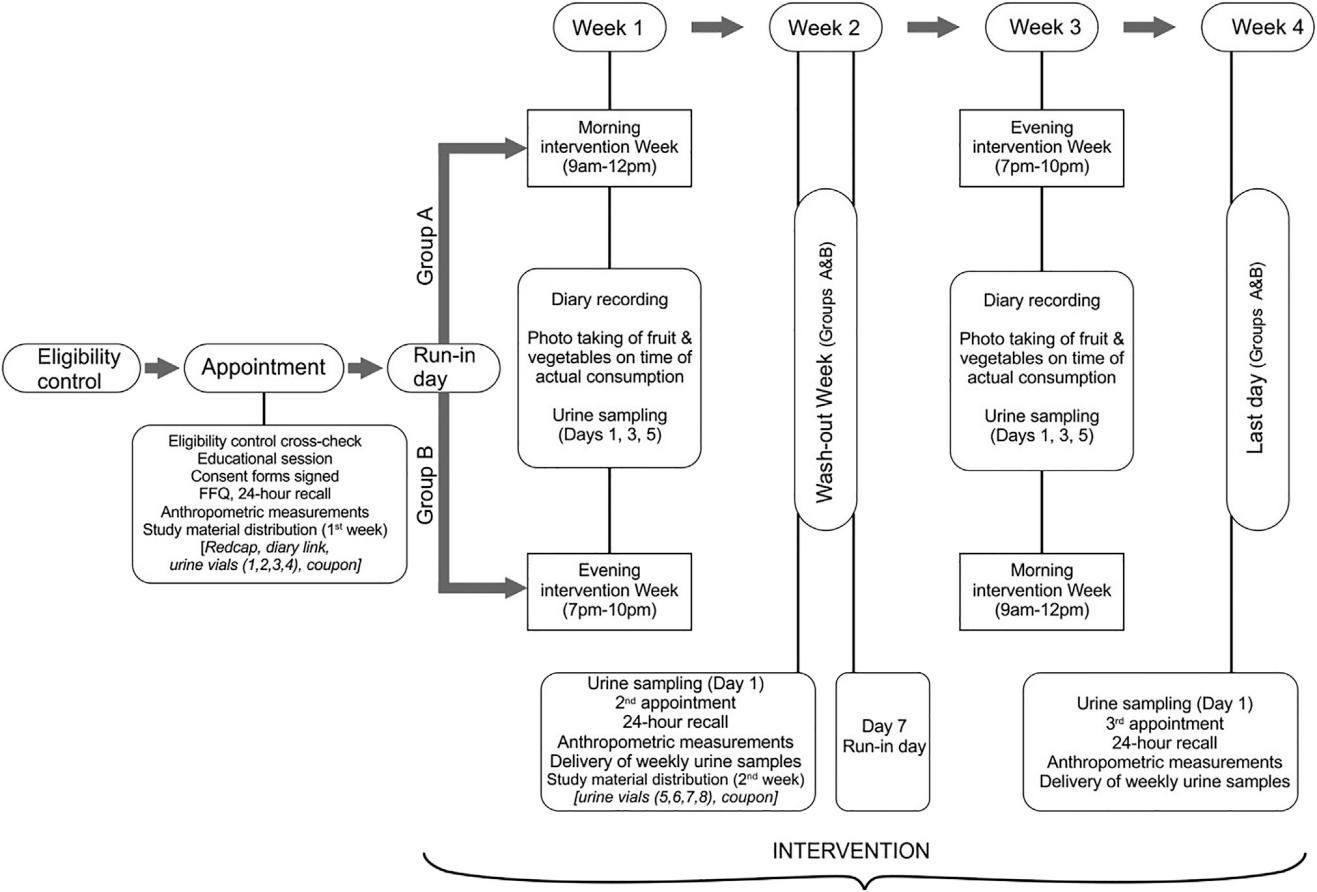
Procedures and timeline of the CIRCA CHEM trial The first morning urine void collection was practiced by all participants during each sampling day, i.e., on the first day of the week, the third day, the fifth day, and the seventh day of each week of the study.

The CIRCA-CHEM study participants were residents living in the urban areas of Limassol/Paphos cities in Cyprus (n = 45) (Figure 1). A total of seven participants were excluded from data analysis, either because they dropped out (n = 4) or because they did not meet the portion compliance criteria (consumed less than 80% of the prescribed fruit and vegetables during both weeks, i.e., n = 98 total fruit/vegetable portions; n = 3). In total, 38 participants were included in the main analysis (Table 1, Figure S1). Most participants were females (74%) with a mean age of 36.8 years. Participants showed overall good compliance to the intervention treatment requirements: fruit and vegetable portion compliance (94 ± 15%), time-restriction compliance (93 ± 20%), and fruits/vegetables portion photo-taking compliance (73 ± 33%) (Table 1).

**Table 1:** Demographics and baseline characteristics of the study population (overall and by group)

	Overall		Group A		Group B		p *value*^a^
	Mean (SD)	n (%)	Mean (SD)	n (%)	Mean (SD)	n (%)	
N		**38**		**21**		**17**	
Male		10 (26.3)		6 (28.6)		4 (23.5)	
Female		28 (73.7)		15 (71.4)		13 (76.5)	
Age (mean (SD))	36.8 (10.7)		38.8 (12.1)		34.3 (8.3)		0.202

**Compliance**							

% Fruit portion consumption compliance (mean (SD))	95 (17)		95 (16)		94 (18)		0.766
% Vegetable portion consumption compliance (mean (SD))	93 (19)		94 (18)		92 (20)		0.098
% Fruit & Vegetable consumption compliance (mean (SD))	94 (15)		95 (14)		93 (17)		0.287
% Time compliance (mean (SD))	93 (20)		94 (16)		91 (23)		**0.08**
% photo compliance (mean (SD))	73 (33)		74.5 (32.7)		72.3 (31.6)		0.766

**Anthropometrics**							

Height (mean (SD)) (cm)			165.3 (6.6)	21	163.5 (6.7)	17	0.425
Weight baseline (mean (SD)) (kg)			70.7 (12.0)	21	64.0 (11.4)	17	0.088
Weight 1st week (mean (SD))			70.4 (11.9)	21	63.7 (11.3)	17	0.092
Weight 3rd week (mean (SD))			70.4 (11.8)	21	63.5 (10.5)	17	0.067
Waist circumference (WC) baseline (mean (SD))			85.5 (10.4)	21	79.7 (10.1)	17	0.09
WC 1st week (mean (SD))			84.9 (10.6)	21	78.8 (9.3)	17	0.082
WC 3rd week (mean (SD))			85.6 (11.2)	21	77.4 (7.8)	17	0.018
BMI baseline (mean (SD))			25.7 (3.0)	21	23.8 (2.9)	17	0.049
BMI 1st week (mean (SD))			25.6 (3.0)	21	23.4 (2.6)	17	0.025
BMI 3rd week (mean (SD))			25.6 (2.9)	21	23.7 (2.7)	17	0.04

**Other**							

Pesticide use (times/week, mean (SD))	1.9 (0.3)	532	1.9 (0.2)	294	1.83 (0.4)	238	**<0.001**
Sleep duration (hours/week, mean (SD))	7.7 (1.3)	532	7.6 (1.2)	294	7.9 (1.3)	238	0.027

**Calories**							

Daily energy intake at baseline (kcal/day, mean (SD))	1712 (735)	105	1795 (843)	60	1599 (548)	45	0.177
% energy (caloric) contribution of the prescribed fruit & vegetable consumption to total daily energy intake (mean (SD))	28.5 (10.9)	70	28.2 (10.3)	40	28.9 (11.7)	30	0.787

**Fruit and vegetable consumption**							

Daily fruit consumption (number of fruits/day, mean (SD))	1.98 (1.3)	38	2.1 (1.4)	21	1.9 (1.2)	17	0.69
Daily vegetable consumption (number of vegetables/day, mean (SD))	2.60 (2.3)	38	2.4 (1.9)	21	2.8 (2.8)	17	0.626
Daily fruit & vegetable consumption (number of fruit & vegetable/day, mean (SD))	4.58 (3.1)	38	4.5 (2.9)	21	4.7 (3.4)	17	0.845
Weekly fruit consumption (number of fruits/week, mean (SD))	13.9 (9.2)	38	14.5 (10.1)	21	13.2 (8.3)	17	0.672
Weekly vegetable consumption (number of vegetables/week, mean (SD))	18.2 (16.1)	38	17.0 (13.2)	21	19.6 (19.5)	17	0.626
Weekly fruit & vegetable consumption (number of fruit & vegetable/week, mean (SD))	32.1 (21.8)	38	31.5 (20.7)	21	32.8 (23.8)	17	0.857

a non-parametric tests.

### Anthropometrics and energy intake

No differences were observed in the baseline anthropometrics between the two groups; weight (Group A: 70.7 ± 12.0 kg, Group B: 64.0 ± 11.4 kg; p = 0.088); body mass index (BMI) (Group A: 25.7 ± 3.0 kg/m^2^, Group B: 23.8 ± 2.9 kg/m^2^; p = 0.049) and waist circumference (Group A: 85.5 ± 10.4 cm, Group B: 79.7 ± 10.1 cm; p = 0.09). Similar results were obtained for the energy intake (Group A: 1795 ± 843 kcal, Group B: 1600 ± 548 kcal; p = 0.177) (Table 1). No statistically significant differences in the anthropometrics were shown at the end of each intervention treatment periods; weight difference (evening: −0.18 ± 1.11 kg, morning: −0.16 ± 2.3 kg; p = 0.96); BMI (evening: −0.04 ± 0.37 kg/m^2^, morning: −0.03 ± 0.29 kg/m^2^; p = 0.879) and waist circumference (evening: 0.29 ± 2.31 cm, morning: −0.42 ± 1.9 cm; p = 0.18). Similarly, no statistically significant differences were seen for the daily energy intake between the two intervention weekly periods (evening: +7 ± 662 kcal, morning: −247 ± 851 kcal; p = 0.19) (Table S1).

### Biomarkers

**Figure 2:**
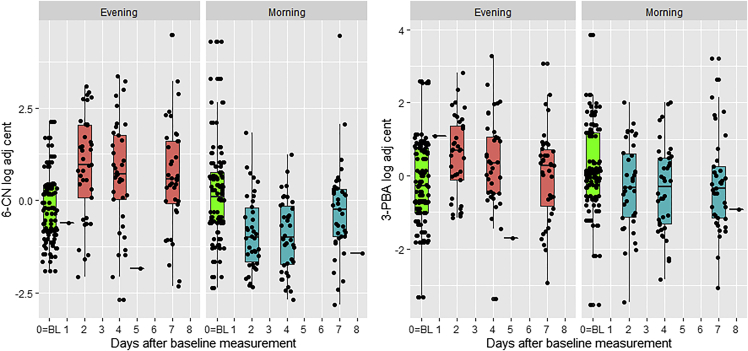
Changes in the biomarkers of exposure to 6-CN (left), 3-PBA (right) from the baseline (green) of each treatment period as a function of days since baseline measurement 6-CN: 6-Chloronicotinic acid (log-transformed, creatinine adjusted, centered); 3-PBA: 3-phenoxybenzoic acid (log-transformed, creatinine adjusted, centered).

Free cortisol measurements were similar between the two treatment periods (morning vs evening weekly intervention, median[IQR] ug/g; 6.3[2.3, 18.8] vs. 6.8 [1.7, 13.4], supplemental information file). The dietary time-restricted intervention resulted in varying levels of the urinary biomarkers of exposure to pesticides, depending on the time of day that the consumption of fruits/vegetables took place (Figure 2). The morning intervention period resulted in a statistically significant decrease (p *<* 0.001) for both pesticide metabolites of 6-CN (beta = −1.99, 95%CI: −2.72, −1.26) and 3-PBA (beta = −1.05, 95%CI: −1.65, −0.45) when compared with the evening intervention period (Table 2). The trends remained unchanged in the sensitivity analysis using only the subjects who were compliant in both treatment periods (n = 33) (supplemental information file). Additionally, we found that males had lower levels of both pesticide metabolites than females. There was a positive association (p *< 0.001*) between 4-HNE and both pesticide biomarkers: 6-CN (beta = 0.11, 95%CI: 0.05, 0.18); 3-PBA (beta = 0.22, 95%CI: 0.15, 0.30) (Table 3).

**Table 2:** Linear mixed-effect models of log-transformed pesticide metabolites and biomarker of effect (4-HNE) as a function of time (# of days of treatment since that week’s baseline, after run-in day), intervention treatment (weekly morning dietary intake of iso-pesticide fruits and vegetables in comparison to the weekly evening dietary intake) and their interaction terms (if p < 0.05), adjusting for the baseline levels of the compounds, and accounting for the repeated measurements

	Coefficient (95% CI)		
	3-PBA (ug/g)	6-CN (ug/g)	4-HNE (ug/g)
Intervention treatment (morning)	−1.05 (−1.65, −0.45)^c^	−1.99 (−2.72, −1.26)^c^	0.12 (−0.25, 0.5)
Time (days) since baseline	−0.11 (−0.20, −0.02)^a^	−0.04 (−0.15, 0.07)	0.01 (−0.05, 0.07)
Sex (males)	−0.86 (−1.39, −0.33)^c^	−0.73 (−1.28, −0.19)^b^	−0.28 (−0.55, −0.01)^a^
Age	0.02 (0.0, 0.04)	0.02 (0.0, 0.05)^a^	0.02 (0.01, 0.03)^c^
Intervention ∗ time since baseline	0.11 (−0.02, 0.23)	0.15 (−0.01, 0.30)	−0.04 (−0.12, 0.03)
Number of samples	206	206	188
Marginal R^2^/Conditional R^2^	0.34/0.50	0.34/0.44	0.16/0.29
ICC	0.24	0.15	0.15

a p value <0.05.

b p value<0.01.

c p value <0.001.

**Table 3:** Linear mixed-effect models of log-transformed 4-HNE as a function of the pesticide metabolite 3-PBA and 6-CN, adjusting for time (# of days of treatment since that week’s baseline, after run-in day), adjusting for the baseline levels of the compounds, and accounting for the repeated measurements

	4-HNE (ug/g) Coefficient (95% CI)	4-HNE (ug/g) Coefficient (95% CI)
Pesticide metabolite (3-PBA)	0.22 (0.15, 0.30)^c^	
Pesticide metabolite (6-CN)		0.11 (0.05, 0.18)^c^
Sex (males)	−0.07 (−0.3, 0.16)	−0.21 (−0.47, 0.06)
Αge	0.01 (0.0, 0.02)^a^	0.02 (0.0, 0.03)^b^
Time since baseline	0.00 (−0.04, 0.04)	−0.02 (−0.06, 0.02)
Number of samples	188	188
Marginal R^2^/Conditional R^2^	0.29/0.35	0.21/0.33
ICC	0.08	0.15

a p value <0.05.

b p value<0.01.

c p value <0.001.

### Nuclear magnetic resonance (NMR) metabolomics profiling

Out of >200 features annotated with the NMR metabolomics platform, a total of 33 were identified and quantified in urine (μM) from the NMR tube (supplemental information, Table S4). Mixed effect models showed that *cis*-aconitate was the sole metabolite associated with the intervention treatment, being higher in the evening period (beta = 0.29, 95%CI: 0.12, 0.47; q = 0.03), while N-methylnicotinamide (NMN) (beta = 0.28, 95%CI: 0.14, 0.42; q = 0.003) and fumarate (beta = 0.22, 95%CI: 0.09, 0.35; q = 0.018) were the sole metabolites that were associated with the biomarkers of exposure to pyrethroids (3-PBA), following multiple testing correction; no NMR metabolite was significantly associated with either 6-CN or 4-HNE, following multiple testing correction (supplemental information, Table S5).

## Discussion

To the best of our knowledge, this is the first RCT that examined a possible chronotoxicity effect of pesticides after adhering to a time-restricted (morning vs. evening) dietary intervention, based on a specific fruit and vegetables consumption pattern (close to the typical “five-a-day" recommendation). The anticipated post-dietary (fruits/vegetables) intake increase in the urinary biomarkers of exposure to pesticides was only observed when the dietary consumption occurred in the evening period (7pm–10pm), but not when the dietary consumption occurred in the morning (9am-12pm). In this dietary intervention, the magnitude of the urinary biomarkers of exposure to either pyrethroid or neonicotinoid pesticides in the evening-based consumption week was significantly higher than those observed in the morning-based intervention period. The 3-PBA and 6-CN biomarkers of exposure to pesticides used in this study are considered validated markers of exposure to pyrethroids and neonicotinoids, respectively, where each of them is a shared metabolite of a number of commonly used parent pesticides of differing structures and relative potency.^39^^,^^40^ This study’s time-differentiated biological phenomenon was consistent with findings from an earlier panel study on the effect of the time of day on urinary biomarkers of exposure to chlorine-based chemicals found in disinfectants (e.g., disinfection by-products, trihalomethanes, THM).^17^ In effect, three independent time series panel studies with cleaning activities using common chlorine-based disinfectants showed that the anticipated post-intervention (household cleaning activities) increase in the biomarkers of exposure to THM was observed only when the intervention-cleaning activities took place in the evening, but not when the intervention took place in the morning/noon hours.^17^

### Chrono-differentiated metabolism of xenobiotics

This diurnal variability in the magnitude of internal biomarkers of exposure and/or downstream effect(s) may be associated with chrono-disrupted or modified human routines, or repetitive behaviors/lifestyles. This chrono-differentiated magnitude of an internal exposure metric has been earlier explained for certain pharmaceuticals on the basis of circadian metabolism. Pharmacokinetic and pharmacodynamics studies have shown the circadian clock regulation of the administered drug’s potency in humans and animal models.^41^ To date, no such human chronotoxicity studies exist for common environmental chemicals (xenobiotics).

Physiologically, human exposures to xenobiotics, like the pesticides may be influenced by the circadian fluctuation of Phase I and II enzyme systems that metabolize such compounds.^42^^,^^43^ The hepatic clock, which also regulates Phase I and II enzymes is mainly conditioned by food intake.^43^ Feeding time appears to be the dominant timing cue for peripheral clocks, such as the hepatic clock.^42^^,^^43^ Phase I enzymes act upon the substrate xenobiotics via circadian-controlled peaks with maxima observed during food ingestion, while Phase II enzymes are typically expressed at different circadian phases, particularly toward the light/inactive phase of animals.^42^ One of the first studies of diurnal variations of pesticide metabolism was performed in male *Drosophila,* demonstrated a clustering of xenobiotic metabolizing gene expression related to pesticides in the daytime, particularly late afternoon implying potential for greater resistance to chemical exposure at that period of the day.^44^ However, unlike insects, mammals have multiple sodium channel isoforms that vary in their biophysical and pharmacological properties, including their differential sensitivity to pyrethroids.^45^ Upon xenobiotic absorption, certain hepatic enzyme systems may be expressed and activated depending on the time of day, dosing, or type of absorbed xenobiotics.

Desynchronized xenobiotics exposures from circadian patterns of hepatic enzyme systems would likely lead to varying degrees of bioactivation, metabolism, and biotoxicity.^46^ Our study showed that *cis*-aconitate was the sole NMR-based metabolite significantly associated with the dietary time-restricted intervention, being higher in levels when dietary consumption took place in the evening period than in the morning period. Aconitate is part of the Krebs citric acid energy production cycle and is involved in energy metabolism. Upon activation of inflammatory stimuli, aconitate acts as a precursor to produce itaconate, a mitochondrial metabolite produced by macrophages regulating immunity at the tissue level.^79^ It has been reported that aconitate cycles at the 8-h frequency, the third harmonic of circadian periodicity, based on data from both mouse livers and cell-autonomous U2 OS systems.^47^ This emphasized the notion that chrono intervention-associated metabolites (e.g., aconitate) would require similar half-lives of elimination to those of the urinary pesticides for their association with the dietary intervention treatment to be detected; indeed, the 3-PBA and 6-CN pesticide metabolites have short biological half-lives, in the order of few hours (∼7–12 h).^48^^,^^49^

### Chrono-disrupted metabolism of xenobiotics and associated chronotoxicity

Positive significant associations were observed between the urinary biomarkers of exposure to pyrethroids (3-PBA and 6-CN) and the marker of lipid-based oxidative damage (4-HNE); 3-PBA was also significantly associated with two NMR-measured metabolites (NMN and fumarate), suggesting a TCA cycle dysfunction. The accumulation of fumarate, a TCA cycle-associated metabolite has been considered a signal of trained immunity, a property of innate immune cells.^80^ Loss of fumarate hydratase enzymatic activity would result in the accumulation of intracellular fumarate, which has been proposed to act as a competitive inhibitor of 2-oxoglutarate-dependent oxygenases, including the hypoxia-inducible factor hydroxylases, thus activating downstream oncogenic pathways.^64^

This positive dose-response association between the pesticides and the marker of oxidative stress (4-HNE) would suggest a type of chronotoxic diurnal window of higher pesticide concentrations that were observed in the evening period, but not in the morning-noon period. This study’s biomarker of effect (lipid peroxidation, 4-HNE) has been implicated with different pathologies, such as metabolic diseases, neurodegenerative diseases and cancers.^50^ Lipid peroxidation may generate reactive oxygen species that induce tissue injury via the formation of lipid peroxidation products, like the 4-HNE.^51^ Under oxidative stress conditions, 4-HNE seems to be the most abundant lipid-derived oxidation product, which at low levels it may promote cell proliferation, while at higher concentrations it induces oxidative DNA alterations and cell apoptosis.^74^ 4-HNE-induced oxidative stress also caused an increase in the expression of GSTA4-4 and CYP2E1 in the mitochondria of PC12 cells.^52^ Based on a study with CYP2E1 deficient mice, 4-HNE was elevated in mice exposed to environmental chemicals when compared to the CYP2E1-knock out mice.^53^ Disinfection chemical byproduct toxicity was mediated by its free radical metabolism by CYP2E1, forming 4-HNE adducts.^53^ Increased CYP2E1 activity was associated with significantly higher 4-HNE levels across various cell types in liver.^54^

The other urinary metabolite (NMN) that was significantly associated with the pyrethroid marker (3-PBA) has been suggested as a biomarker of peroxisome proliferation (PP) and it has been accompanied by a marked increase in the size and number of hepatocellular peroxisomes in rats.^56^ The PP response (hepatomegaly, increased peroxisome enzymes) is due almost exclusively to the PPARa, which is highly expressed in cells with active fatty acid oxidation capacity, including hepatocytes.^56^ Overexpression of hepatic PPARα in rodents led to hepatic peroxisome proliferation and expression of peroxisomal acyl-CoA oxidases (ACOX1), leading to oxidative stress and damage.^63^ Urinary metabolomic profiling in two rodent models of diabetes and human patients with type II diabetes confirmed the relative increase in NMN levels and concomitant perturbations in tryptophan (precursor of NMN) and nucleotide metabolism;^57^ NMN-associated perturbation in nucleotide metabolism has been previously linked with liver peroxisome proliferation.^57^ Pyrethrins were among those few pesticides that induced PPARα agonistic activity *in vivo*, as well as *in vitro*, out of a total of 200 pesticides initially tested.^58^ Well known examples of peroxisome proliferators that impinge upon the PPAR nuclear receptors are the fibrate drug, phthalates, and PFAS, just to name a few.^59^ Such xenobiotics may interact with PPARs, triggering biochemical pathways that affect multiple biological systems and disease processes, including coronary artery disease risk, metabolic syndrome, kidney disease and breast cancer.^60^ It is well known that the core clock dictates the rhythm of organ-based transcription factors and nuclear receptors (e.g., PPAR) that in turn could regulate the cyclic expression of xenobiotics-processing genes/enzymes.^55^ PPARs have been shown to function as master regulators of the adaptive response to xenobiotics via regulation of Phases I, II, and III enzyme systems. In human hepatocytes, PPARα activates members of CYP1A, CYP2A, CYP2B, CYP2C, CYP2E, CYP2J, and CYP3A subfamilies.^61^ For example, PPAR-γ positively regulated CYP2A5 activity in Hepa-1C1c7 cells, showing that the nuclear receptor PPAR-γ was a transcriptional activator of CYP2A5, and its rhythmic expression contributed to the circadian expression of CYP2A5.^62^

It is worth noting that the observed type of toxicity (oxidative stress) and its particular chrono-window (evening period) may not be the sole manifestation of the dietary pesticides’ human toxicity effects. The absence of a post-dietary intake increase in the magnitude of the biomarkers of exposure to pesticides during the morning-noon hours does not exclude the possibility that a different type of toxicity may be conferred. The general notion is that increased metabolism of pesticides may actually reduce their toxicity, if the parent compound is more toxic than the metabolite. This may be particularly relevant for acute toxicity effects of pyrethroids on mammals, which would be enhanced by compounds that limit parent pesticide metabolism by inhibiting hepatic esterases and monooxygenases.^65^^,^^66^ Hepatic tissues indeed sustain high levels of pyrethroid-metabolizing enzymatic activity, catalyzing the principal biotransformation pathways for parent pyrethroids. Despite their lipophilicity, pyrethroids do not bioaccumulate in lipid-rich tissues, because within a few hours they are readily metabolized by hydrolases and cytochrome P450-dependent monooxygenases in the liver and other tissues.^65^^,^^66^ The pesticide metabolites (e.g., 3-PBA) derived from the ester cleavage products of permethrin, cypermethrin, deltamethrin, fenpropathrin, and fenvalerate, are generally considered being of equal or lower acute toxicity to mammals than the parent pyrethroids.^65^^,^^66^ In animal studies, these two considerations imply that metabolism effectively limits the expression of the acute toxicity of pyrethroids; the metabolites of pyrethroid biotransformation typically exhibit lower acute toxicity than the parent pyrethroids.^65^^,^^66^ However, the assumption that the pyrethroid pesticide metabolites are less toxic than the parent compounds may not be strictly valid for lower pesticide doses, like those encountered in this study. Resembling a five-a-day fruits and vegetables daily intake scenario, this study’s pesticide exposures were closer to those encountered in a lower level and chronic pesticide exposure scenario. Indeed, a widespread low-level pesticide exposure for the European populations has been typically the case with 63 and 45% of fruit/tree nuts and vegetable samples, respectively, having detectable pesticide levels ≤ maximum residue levels (MRL), and 4.0 and 5.6% of fruit/tree nuts and vegetable samples, respectively, having pesticide levels > corresponding MRL.^67^ Further, this study’s pesticide metabolite (3-PBA) has been ubiquitously detected in urine of U.S. and European populations.^48^^,^^68^ The 3-PBA is one of the validated pyrethroid pesticide metabolites that has been extensively used as the main pesticide exposure metric in epidemiological studies. The higher the urinary 3-PBA metabolite levels, the higher the pyrethroid human health risk; for example, 3-PBA inclusion in the list of pyrethroid markers for the US National Health and Nutrition Examination Survey has resulted in the evaluation of numerous epidemiological associations of pyrethroid exposure with chronic disease outcomes in adults.^69^

The difference of 10–12 h in urine sampling times between the two treatment periods would not be held responsible for the observed chrono-differentiated treatment effect in this study. First-morning urine voids were intentionally collected for all repeated samplings, instead of collecting urine voids with different sampling time stamps in the day (e.g., if based on the treatment period). This was *a priori* decided in order to standardize sampling time stamps to the external *zeitgeiber* of the circadian day/night cycle variation by collecting only the first morning voids and correcting for urinary creatinine levels. Moreover, the studied pesticides’ half-lives were relatively short (∼7–12 h).^48^^,^^49^ As such, the external pesticide dosing was daily repeated for 7 days in each weekly treatment period, leaving no room for the pesticides’ levels to return to baseline, as this would be the case for a single pesticide dose experiment. Thus, an apparent equilibrium internal dose would be established during each weekly treatment period that the longitudinal design of the trial allowed us to observe. It has been well established that for repeated exposures to nonpersistent chemicals, like the studied pesticides, the amount in a given biological sample would likely represent the average exposure being continuously replenishing the biomarker in the blood, while the urinary elimination of the studied pesticide would reach a steady state.^70^ A similar intervention of an early time-restricted feeding schedule (eTRF) (6-h feeding period, with dinner before 3p.m.) reduced fasting insulin and oxidative stress levels relative to those of a control schedule (12-h feeding period, dinner in evening 7p.m.) using a 3-h OGTT test taking place in the morning for both eTRF and control schedule treatments.^71^

### Implications of chronotoxicity for personalized prevention initiatives

The observed chrono-differentiated internal response of pesticides to a time-restricted dietary treatment would be highly relevant for the personalized medicine initiatives on both sides of the Atlantic Ocean. The *All of Us* research program led by the U.S. National Institutes of Health was recently set up based on the initiative of President Obama’s Precision Medicine Initiative Working Group (2015), while the European Council conclusions put forth the legal framework on personalized medicine initiatives in Europe (2015/C 421/03). Personalized medicine or personalized prevention may study differences in people’s genes, environments and lifestyles, hence, they can provide knowledge about differentiated health risk profiling and population stratification, and how such information may be used to improve people’s health.^82^ Tailoring medical models to the right person or risk subgroups and delivered at the right time may be of utmost importance in personalized medicine or personalized prevention initiatives; such models may choose to focus on early-stage biomarker discovery and/or on disease prevention and prediction schemes.

From a methodological and study design perspective, the chrono-differentiated metabolism effect could find use in environmental epidemiology studies. Assuming diurnal or circadian effects on the biomarker of exposure levels, uncontrolled biospecimen collection times in the day or individual behaviors might lead to nondifferential exposure misclassification likely leading to bias of the measure of association toward the null.^72^ This effect might be more evident with single spot biospecimen samples in a day, highlighting again the importance of choosing appropriate time windows for specific exposure/dose metrics.

The crossover study design and its randomized nature are the strengths of this RCT, including the coupling of biomarkers of exposure and effect with the detection and quantification of known urinary metabolites using NMR metabolomics platforms. The high compliance of the RCT was supported by frequent communication protocols with the participants and user-friendly electronic tools to facilitate sharing of pictures and taking meals at prescribed times of day. An iso-pesticide feature was achieved by narrowing down the pesticide residue content variance of foods used in this trial; this was achieved by choosing a single market for all study participants, ensuring that all fruits/vegetables were coming from the same producer(s) throughout the study period. All foods used by participants were fresh and seasonal and they were collected from the single market store by each participant on a weekly basis. Further, a run-in day (no fruit and vegetable consumption) that chronologically preceded each weekly period of the study, contributed to having a narrow and small baseline signal for the urinary pesticides of interest. Despite the above precautions, some variation in the pesticide content of the foods consumed and/or individual exposures to non-dietary pesticide sources (e.g., insecticide use) was anticipated. Non-dietary exposure to pesticides at least once for both study weeks was reported only by 7 participants. Hence, the inclusion of repeated measures (7–8 time points per subject) and the use of proper statistical models (mixed effect models) allowed us to better capture the within- and between-subject variability in the study outcomes.

It is evident that more data is needed to infer chronotoxicity windows of differentiated health risks associated with a systematic time-restricted dietary habit. The field of human chronotoxicity/chronometabolism of environmental chemicals (e.g., food contaminants) is largely unexplored, with most of the chronotoxicity developments referring to research on pharmaceutical drugs in the clinical setting (e.g., cancer chronotherapy).^73^ More studies are needed to investigate the impact of controlling times of exposure to xenobiotics, such as food contaminants, or other environmental stressors on disease risk prevention and prediction using a personalized medicine approach. More data is warranted to better understand the chronotoxicity-based health effects of xenobiotics, like pesticides, by considering the circadian or 24-h, or other cycle-based diurnal disruptions (e.g., night shift work) and interfacial desynchronizations between the external time and the internal circadian time, likely causing excess toxicity risk.^46^

### Limitations of the study

Limitations were the small sample size of the trial and the adult population’s characteristics, which may limit the generalizability of the results. Further, the targeted profiling of the chosen NMR metabolomics platform only allowed us to capture a very small percentage of the total urine metabolome (>110K compounds), calling for other high-throughput metabolomics protocols (e.g., mass spectrometry). Another limitation was the lack of direct estimation of pesticide metabolizing enzymes or measurements of other biomarkers of oxidative damage. This work is a starting point for the investigation of chronotoxicity window(s) associated with chrono-disrupted metabolism of food contaminants that may find use in personalized medicine and personalized prevention initiatives.

## STAR★Methods

### Key resources table

**Table:** 

REAGENT or RESOURCE	SOURCE	IDENTIFIER
**Software and algorithms**		

NutriSoft®	NutriSoft software	http://www.cheapsoftcyprus.com
R version 4.1.2 (2021-11-01)	R Web Site	R Core Team, 2017. R: A Language and Environment for Statistical Computing. Vienna, Austria: R Foundation for Statistical Computing. Available at: www.r-project.org/.
RStudio "Ghost Orchid" Release (8b9ced18, 2021-11-08)	Rstudio website	RStudio Team, 2015. RStudio: Integrated Development Environment for R. Available at: www.rstudio.com/

### Resource availability

#### Lead contact

Further information and requests for resources should be directed to and will be fulfilled by the lead contact, Konstantinos C. Makris, PhD (konstantinos.makris@cut.ac.cy).

#### Materials availability

This study did not generate new unique reagents.

### Experimental model and subject details

#### Trial Oversight

The CIRCA CHEM trial was an investigator initiated 2x2 cross-over, randomized controlled chronotoxicity pilot trial and registered with ClinicalTrials.gov (NCT04177030). The authors assume responsibility for the accuracy and completeness of the data and analyses, as well as for the fidelity of the trial. The trial protocol was approved by the Cyprus National Bioethics Committee (EEBK/EΠ 2019/103). The trial was conducted during August-December 2020, and it was performed in accordance with the principles of the Declaration of Helsinki. Funding sources had no role in the design of this study, and did not have any role during its execution, analyses, interpretation of the data, or decision to submit results.

#### Trial population

The trial eligibility criteria were the following: Healthy adults aged between 18-60 years old, with a BMI of 18–30 kg/m^2^ and being permanent residents in the Republic of Cyprus for the past 5 years. Moreover, systematic organic fruit and vegetable consumers (more than 80% of their weekly usual fruit and vegetables consumption), people with food allergies especially on the indicated fruit and vegetables, people with sleep disorders (who systematically sleep less than 6 hours per day or who systematically have more than 2 involuntarily sleep interruptions with difficulty getting back to sleep within the night), shift workers (i.e. doctors, nurses, police and fire-department officers, etc.) and pregnant and lactating women were excluded from the trial. Good health status was assessed with questions regarding the presence of any chronic or other disease or from the systematic intake of prescribed medication. If the participant met all eligibility criteria, then, an informed consent was signed in order to proceed with the study protocol.

#### Randomization and masking

A total of 45 eligible adults were *a priori* randomly allocated by the lead researcher in blocks of five to two groups (A and B) that differed in the sequence of the treatments (CONSORT flow diagram, Figure S1); both groups were asked to adhere to the same fruits and vegetables daily dietary scheme in two different time-restricted windows of the day (morning and night) for a period of one week for each time-restricted intervention period. Group A (n=24) was assigned to start with the morning intervention (9am-12pm) for the first week and then moved to the evening intervention week (7pm–10pm), whereas Group B (n=21) started the evening intervention first and then moved to the morning intervention period. In-between the two intervention weeks, a wash-out week intervened in which participants were asked to return to their usual eating habits with the only limitation being that participants abstained from organic fruit and vegetables consumption. For an one-day period prior to the commence of each intervention week, participants were asked to completely abstain from the consumption of fruit and vegetables (run-in-day) (Figure 1).

The intervention treatment in which participants were asked to adhere during the weekly restricted time-windows included the consumption of the same amount and type of fruit and vegetables that were purchased from the same local market store by all participants. The fruit and vegetables selected for the trial were an orange, an apple, a pear and a banana (1 medium size for each of the fruit), a tomato (1 medium size tomato), two cucumbers (2 small) and lettuce (1 cup of chopped lettuce), counting for 4 and 2 dietary equivalents, respectively. The number of fruit and vegetables was based on the joint WHO/FAO^24^ recommendation for the general population encouraging the consumption of “5-a-day”. The type of fruit and vegetables selection was performed for market availability reasons throughout all seasons of the year, and based on the assumption that these products are usually well accepted by the general population in terms of taste preference and tolerance. A single fruit/vegetables market store was the sole provider of the fruits and vegetables for the study participants, who would come to the store and select the weekly food items with a coupon. Each participant was given a coupon for receiving for free the week’s total portions of fruit and vegetables from the collaborating market store. We selected to collaborate with a local market store to ensure that all participants consumed products from the same producer(s) throughout the study period, and to better control the variation in pesticide residue content of foods. The vast majority of foods in the study were locally produced and the rest came from Greece during the study period.

Participants were strongly advised to confine their daily fruit and vegetables dose only during the assigned time-windows for each week. During the meeting with the lead researcher, all participants were advised to wash the fruits and vegetables with water before consumption (common practice), and emphasis was placed on their consumption with the skin, where appropriate. Besides the time-restricted dietary treatment each day of the week, participants were asked to otherwise keep to their usual dietary habits. They could consume other food throughout the day in and out the restricted time-windows indicated; participants were however asked not to consume coffee and alcohol during the three-hour restriction slot. They could still consume up to two portions of coffee and two-portions of alcohol within the day outside the indicated restriction hours. Also, participants were asked to strictly avoid lemon, lemon juice, lime juice, grapefruit and grapefruit juice and any other fruit juice consumption during the two intervention weeks in order to avoid interference with regular liver enzymatic activity.^25^

### Method details

#### Anthropometrics and food frequency questionnaire

Upon participant’s written consent, a dietary assessment was performed using a validated and adjusted for the Cypriot population Food Frequency Questionnaire (FFQ) (supplemental information file).^26^^,^^27^^,^^28^ The recorded information was used as a reference point with regards to participants’ general dietary habits (for example the average intake of fruit and vegetables, average water intake, etc.). Additionally, the lead researcher performed a 24-hour dietary recall (24-HDR) (SI.2) taking detailed information on what the participant consumed (food and beverages) during the previous day. Data collected with the 24-HDR during each appointment was used to compare differences in the diet of participants by estimating their caloric intake, comparing it between baseline and treatment periods (supplemental information file). The important customized dates of the trial were highlighted in a diary given to each participant (run-in-days, intervention days, urine sampling dates, appointment dates).

Anthropometric measurements were taken during eligibility control. Weight (Wt) and height (Ht) measurements were taken using an electronic scale (Wunder Ra200®, Italy). Participants were measured with light clothing on and without shoes. Body mass index (BMI) was calculated with the equation of Wt/Ht^2^. The waist circumference (WC) was measured at the approximate midpoint between the lower margin of the last palpable rib and the top of the ileac crest with the use of a plastic measuring tape (200cm) following the WHO STEPS protocol.^29^

A unique participant id allowed for the digital management of all collected data for the duration of the trial using the *RedCap®* app and the reminder text messages automated to be sent to participant’s cell phone, using the *Twilio* app. The electronic diary app was used as daily diary with information regarding fruit and vegetable portion and time of consumption compliance, water and beverages consumption and other lifestyle information (physical activity, occasional medicine and food supplement usage, pesticide usage/ exposure, smoking habits). The participant received morning mobile text reminders for completing the electronic diary and texts in the evenings before next day morning urine collection. The electronic diaries were requested to be completed each day for the previous day (S4). All electronic diaries were completed successfully, because the application allowed the participant to continue with completing a following day, only if the previous day’s diary was fully completed. Participants were also asked to take a picture the fruit and vegetables portions each day during or before consumption and shared electronically with the researcher via *Viber®* app. A follow-up appointment (second appointment) was scheduled. The duration of the first appointment was approximately an hour.

#### Urine sampling

Participants were advised to collect first morning urine voids as soon as they woke up. First morning urine voids were collected on specific days of each intervention week (Figure 1). In total, 4 urine samples were collected for the first week and another four urine voids on the third week (the wash out period lasted one week). The first urine void of each week served as the baseline of each intervention week. Urine vials were temporarily stored in the participant’s freezer (−20°C) until transferred to laboratory facilities for storage at −80°C.

Participants were advised to avoid the consumption of any organic fruit and vegetables during the wash-out week and remember that the last day of the week would serve as a second run-in day; thus, they should totally avoid the consumption of fruit and vegetables for 24 hours prior the first day of the second intervention week, in which participants would transition to the opposite time-restricted window (morning to evening or evening to morning). Participants were also reminded that they would again have to complete the electronic diaries and to share time compliance photographs with the lead researcher using the mobile application. The timeline of the study is shown in Figure 1.

#### Outcomes

Per the trial protocol, the primary outcomes were: i) the urinary biomarkers of exposure to pyrethroid pesticides (3-phenoxybenzoic acid, 3-PBA), and neonicotinoid pesticides, (6-chloronicotinic acid, 6-CN), after adhering to a time-restricted (morning vs evening) fruit and vegetable dietary intervention pattern, and ii) changes in the urinary metabolomics profile following the dietary intervention pattern. Additionally, we evaluated the association between the biomarkers of exposure to pesticides with those of the biomarker of effect, i.e., the biomarker of oxidative damage-4-hydroxynonenal (4-HNE). The secondary outcomes were changes in the anthropometric measurements of weight (Wt), body mass index (BMI) and waist circumference (WC) within the two different time-restricted periods.

#### Biomarker measurements

We measured two pesticide metabolites in urine samples: 3-PBA, a metabolite of pyrethroid pesticides, and 6-CN, a metabolite of neonicotinoid pesticides. The concentrations of 3-PBA and 6-CN in urine were measured using a GC (Agilent 7890A) coupled with a triple quadrupole mass spectrometer (Agilent 7000B, Agilent Technologies, Waldbronn, Germany) (GC–MS/MS), based on our lab’s published protocol.^30^ The limits of detection (LOD) and limits of quantification (LOQ) (in parenthesis) were: 0.075 (0.226) ng/L for 6-CN, and 0.049 (0.146) ng/L for 3-PBA. Urinary creatinine was measured using the colorimetric Jaffé method.^31^ The 4-HNE measurements were obtained with published bioanalytical protocols using a GC (Agilent Intuvo 9000) coupled with a quadrupole mass spectrometer (5977B MSD, Agilent Technologies, Waldbronn, Germany).^32^^,^^33^ The urinary free cortisol measurements were conducted on a GC (Agilent 7890A) coupled with a triple quadrupole mass spectrometer (Agilent 7000B, Agilent Technologies, Waldbronn, Germany). following an adaptation of a bioanalytical protocol.^81^

#### ^1^H-NMR metabolomics

Metabolite concentrations were measured in urine using the high-throughput proton Nuclear Magnetic Resonance (1H-NMR) metabolomics platform at the Centre for Omic Sciences, joint Unit of the University Rovira i Virgili and the Technology Centre of Catalonia EURECAT. ^1^H-NMR spectra were recorded at 300K on an Avance III 600 spectrometer (Bruker®, Karlsruhe, Germany) operating at a proton frequency of 600.20 MHz using a 5mm PBBO gradient probe. All acquired ^1^H NMR spectra were compared to references of the pure selected compounds with the metabolic profiling AMIX spectra database (Bruker), HMDB, Chenomx and built in-house databases for metabolite identification. In addition, metabolites were confirmed by ^1^H–^1^H homonuclear correlation (COSY and TOCSY) and ^1^H–^13^C heteronuclear (HSQC) 2D NMR experiments and by correlation with pure compounds run in-house, when needed. After pre-processing, specific ^1^H NMR regions identified in the spectra were integrated (see supplemental information). Details on the methodology of the NMR platform can be found in the supplemental information file.

### Quantification and statistical analysis

The analysis of the population consisted of all participants who completed the trial, excluding those with weekly portion compliance of less than 80% (or more than 120%) of the prescribed total weekly fruit and vegetables portions (i.e., <39 fruit and vegetables portions or >59 during both intervention weeks, n=3); participants who dropped-out at any time of the study and participants who delivered less than 80% of the requested urine samples were also excluded (n=4).

The baseline characteristics were compared between the two groups, A and B, including 38 participants who initiated or partially completed the study, and eventually 38 participants were included in the main analysis. Categorical variables were described with sample size and percentages and compared by chi-square test. Approximately normally-distributed continuous variables were described with means and standard deviations (SD), and compared by t-test, while non-normal continuous variables with medians and interquartile ranges (25^th^–75^th^ percentiles) were compared by the Wilcoxon rank sum test.

The mean daily energy intake was calculated based on the calories of each item described during the 24 dietary hour recalls (baseline, end of each study week) using a software application (*NutriSoft®*) that contained food libraries from Cypriot, Greek and international foods. Differences in caloric intake were compared with a paired t-test for: i) end of first week compared to baseline and ii) end of second week compared with end of first week between the two time-restricted interventions. The anthropometric differences were calculated in the same way.

Pesticide biomarker values <LOD were imputed with regression on order statistics (ROS),^34^ if they contained ≥20% values below detection. All biomarker data were corrected for urine dilution (biomarker mass per gram of urinary creatinine) prior to statistical analysis.

Mixed effect linear regression models were used to account for the effect of the type of time of day intervention (morning vs. evening) on each biomarker of exposure to pesticides (6-CN and 3-PBA) and on the biomarker of effect (4-HNE). All models included repeated measures within person and the urine samples collected within each intervention week. Continuous variables, other than time (days since baseline measurement) were centered at the population means. Linear mixed effect models were fitted for the pesticides and the 4-HNE biomarkers (creatinine-corrected, log-transformed, centered). Models included baseline biomarker measurements, time (i.e., urine sampling day, calculated as number of days since baseline), sex, age, and the interaction of the type of intervention and time (urine sample, number of days since baseline). Additionally, another model was used to check the association between the biomarker of effect (4-HNE) with each one of the pesticide metabolites (6-CN and 3-PBA). A sensitivity analysis that filtered out participants with compliance <80% in either one of the 2 intervention periods was conducted with the remaining 33 participants using the above mixed effect linear regression models.

#### Metabolomics data analysis

The main objective of this statistical analysis was to examine associations between NMR-based urinary metabolites and: i) the intervention phase period (phase of morning vs. evening), ii) the biomarkers of exposure to pesticides and iii) the biomarker of effect (oxidative damage, 4-HNE). Urinary metabolomics profile data processing followed the protocol by Konstantinou et al.^35^ The detected and quantified metabolites from the NMR pre-processing stage were log-transformed, centered (based on population means), and scaled (using the standard deviation), including the biomarkers of exposure to pyrethroids (3-PBA) and neonicotinoids (6-CN values <LOD were imputed with LOD/2) and the 4-HNE. The first set of mixed effect models (i) below, included an interaction term of intervention x time since baseline that was subsequently dropped, if it did not meet the threshold of p-value ≤ 0.05. In effect, the following three sets of metabolomics-based linear mixed effect models were prepared:

i) Metabolite∼baseline of each period+ intervention+BMI+age+sex+creatinine+time since baseline+ intervention∗time since baseline +(1|SubjectID)
ii) Pesticide∼ baseline of each period+Metabolite + BMI+age+sex+creatinine+time since baseline + (1|SubjectID)
iii) 4HNE ∼ baseline of each period +Metabolite + BMI+age+sex+ creatinine+time since baseline + (1|SubjectID)

All associations were corrected for false-discovery rate of 0.05.^36^ Statistical tests and confidence intervals were two-sided, and 95% confidence intervals were used. Statistical analysis was conducted in R version 4.1.2 (2021-11-01)^37^ and RStudio "Ghost Orchid" Release (8b9ced18, 2021-11-08).^37^^,^^38^

## Data Availability

All original code has been deposited at Zenodo and is publicly available as of the date of publication. The DOI is: https://doi.org/10.5281/zenodo.7520917. De-identified human data have been deposited at Zenodo-Github. They are publicly available as of the date of publication. Any additional information required to reanalyze the data reported in this paper is available from the lead contact upon request.

## Supplemental information

Document S1. Figure S1, Tables S1–S6, and Data S1–S4

Data S5. Sensitivity analysis, related to Figure 2

Data S6. Descriptives, related to Table 1

Data S7. NMR metabolomics, related to Figure 2

## Footnotes

Supplemental information can be found online at https://doi.org/10.1016/j.isci.2022.105847.
