# Supplementary material for: A time differentiated dietary intervention effect on the biomarkers of exposure to pyrethroids and neonicotinoids pesticides
Source: iScience. 2022 Dec 22;26(2):105847. doi: 10.1016/j.isci.2022.105847 (PMC9874006; doi:10.1016/j.isci.2022.105847)
Supplement: Document S1. Figure S1, Tables S1–S6, and Data S1–S4 [file mmc1.pdf]

iScience, Volume *26*

Supplemental information

A time differentiated dietary intervention effect on the biomarkers of exposure to pyrethroids and neonicotinoids pesticides

Elina Ioannou, Stavros Oikonomou, Nikolaos Efthymiou, Andria Constantinou, Thibaut Delplancke, Pantelis Charisiadis, and Konstantinos C. Makris

Supplemental information

A time differentiated dietary intervention effect on the biomarkers of exposure to pyrethroids and neonicotinoids pesticides.

Elina Ioannou, Stavros Oikonomou, Nikolaos Efthymiou, Andria Constantinou, Thibaut Delplancke, Pantelis Charisiadis, and Konstantinos C. Makris

Supplementary Material

1. Supplementary Tables and Figures 3
   1.1 Fig. S1 Flowchart of the CIRCA-CHEM trial (Related to Table 1) 3
   1.2 Table S1 (Related to Table 1) Measurements differences in anthropometrics and energy (caloric) intake between the two interventions (evening vs morning). 4
   1.3 Table S2 (Related to Figure 2): Comparison of the medians of the pesticide metabolites, creatinine and the biomarker of oxidative stress during both treatments (evening vs morning)... 4
   1.4 Table S3 (Related to Figure 2): Differences in the concentrations between baseline measurement based on intervention type 4
   1.5 Table S4 (Related to Figure 2) Quantiles of the biomarkers concentrations (μg/L, overall, baseline, treatment) 6
2. Data S1: Food Frequency Questionnaire (FFQ) as validated in Greek (related to STAR Methods) 9
3. Data S2: 24-hour Dietary Recall (24-DHR) in Greek language of the study participants (related to STAR Methods) 22
4. Data S3: RedCap® online diary (related to STAR Methods) 24
6. Data S4: NMR Methodology (related to STARS Methods) …………………………………………………………………………..28
7. Data S5: Sensitivity analysis (as excel file) (related to Figure 2) ………… ……………….
8. Data S6: Descriptives (as excel file) (related to Table 1) ………………………………….
9. Data S7: NMR metabolomics (as excel file) (related to Figure 2)

1. Supplementary Tables and Figures

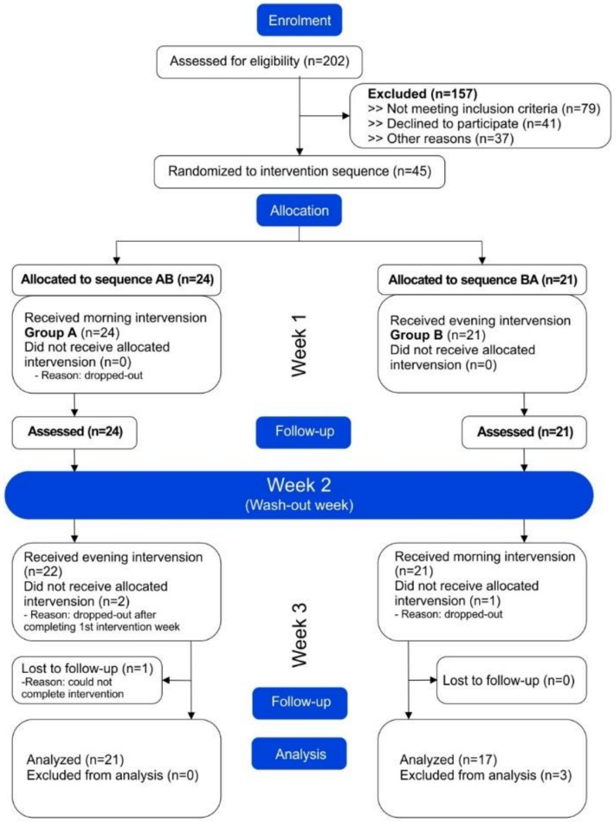

Dawn et al. CONSORT flow diagram for crossover trials. BMJ 2019, 366: bmj.i4378

1.1 **Fig. S1 Flowchart of the CIRCA-CHEM trial** (Related to Table 1)

1.2 **Table S1** (Related to Table 1) Measurements differences in anthropometrics and energy (caloric) intake between the two interventions (evening vs morning).

	**N**	**Evening**	**N**	**Morning**	**p-value**
Wt (mean (SD))	37	-0.18 (1.11)	37	-0.16 (2.3)	0.96
BMI (mean (SD))	36	-0.04 (0.37)	36	-0.03 (0.29)	0.884
WC (mean (SD))	34	0.29 (2.3)	34	-0.42 (1.91)	0.181
Energy intake difference (mean (SD))	35	7.00 (661.99)	35	-247.03 (851.27)	0.19

Wt: weight in kilograms (kg); BMI: Body Mass Index in kilograms (meters)^2 (kg/m$^{2)}$; WC: waist circumference in centimeters (cm); Energy intake difference in calories (kcal)

1.3 **Table S2** (Related to Figure 2): Comparison of the medians of the pesticide metabolites, creatinine and the biomarker of oxidative stress during both treatments (evening vs morning).

**Biomarker**	**Overall**	**Evening**	**Morning**
	Median [25th-75th percentile]	Median [25th-75th percentile]	Median [25th-75th percentile]
n	273	134	139
3-PBA (μg/g Cr)	1.99 [1.16, 3.99]	2.39 [1.22, 4.43]	1.67 [1.09, 3.52]
6CN ((μg/g Cr)	0.09 [0.03, 0.18]	0.13 [0.03, 0.31]	0.03 [0.03, 0.11]
Creatinine (g/L)	1.38 [0.90, 2.13]	1.36 [0.84, 2.09]	1.38 [0.94, 2.31]
4HNE (μg/g Cr)	138.71 [99.40, 208.56]	131.35 [95.04, 207.29]	140.85 [109.13, 217.29]
Cortisol (μg/g Cr)	6.67 [2.04, 14.8]	6.28 [2.34,18.8]	6.79 [1.72, 13.4]

1.4 **Table S3** (Related to Figure 2): Differences in the concentrations between baseline measurement based on intervention type

	**Overall**	**Evening**	**Morning**

N	187	88	99
6CN_baseline (median [IQR])	0.03 [0.03, 0.11]	0.03 [0.03, 0.11]	0.03 [0.03, 0.10]
6CN_treatment (median [IQR])	0.10 [0.03, 0.21]	0.20 [0.09, 0.44]	0.03 [0.03, 0.11]
3-PBA_baseline (median [IQR])	1.58 [1.19, 2.79]	1.54 [0.83, 2.75]	1.58 [1.26, 2.79]
3-PBA_treatment (median [IQR])	2.07 [1.14, 4.49]	2.57 [1.44, 4.87]	1.78 [0.97, 3.61]
4-HNE_baseline (median [IQR])	156.42 [105.21, 229.23]	167.78 [104.07, 233.27]	153.91 [105.73, 192.62]
4-HNE_treatment (median [IQR])	133.74 [99.27, 208.60]	128.46 [94.54, 202.18]	138.71 [110.85, 226.99]
Cor_baseline (median [IQR])	12.07 [5.04, 23.26]	12.17 [4.65,23.48]	10.28 [5.28, 22.69]
Cor_treatment (median [IQR])	9.87 [2.80, 22.96]	9.41 [2.65, 19.93]	10.32 [3.13,24.71]

1.5 **Table S4** (Related to Figure 2) Quantiles of the biomarkers concentrations (μg/L, overall, baseline, treatment)

	Percentiles						
	min	5th	25th	50th	75th	95th	max
**Pesticide metabolite**	**Overall**						
6-CN	0.03	0.03	0.03	0.09	0.19	0.82	4.22
3-PBA	0.17	0.35	1.16	2	3.99	11.81	32.85
Creatinine	0.06	0.37	0.84	1.31	2.09	3.69	7.78
4-HNE	54.56	68.76	99	136.14	200.42	353.49	733.51
Cor	0.09	0.09	3.13	9.93	23.15	49.96	82.61
	**Baseline**						
6-CN	0.03	0.03	0.03	0.03	0.11	0.22	0.6
3-PBA	0.17	0.31	1.19	1.61	2.81	9.24	11.17
4-HNE	56.18	70.74	102.68	153.17	225.71	425.49	733.51
Cor	0.09	0.09	4.65	11.38	23.04	50.40	82.41
	**Treatment**						
6-CN	0.03	0.03	0.03	0.1	0.23	1.01	4.22
3-PBA	0.19	0.49	1.14	2.21	4.5	13.55	32.85
4-HNE	54.56	68.68	98.51	131.7	194.27	341.5	648.71
Cor	0.09	0.09	2.67	9.61	23.11	46.28	74.34

1.6 **Table S5:** Mixed effect models using the NMR metabolomics dataset showing only the significant associations (original p values and q values after FDR correction) (related to Figure 2)

Response	Predictor	estimate	p.value	p.adjust	conf.low	conf.high
cis-Aconitate	Phase:Evening	0.2948843	0.0009641	0.0298871	0.1212747	0.4684939
Citrate	Phase:Evening	0.1974765	0.0168607	0.2613415	0.0358850	0.3590680
Trigonelline	Phase:Evening	0.2042517	0.0380077	0.3927462	0.0114108	0.3970926

Response	Predictor	estimate	p.value	p.adjust	conf.low	conf.high
3PBA	N-Methylnicotinamide	0.27775	0.0001	0.003312	0.13901	0.41648
3PBA	Fumarate	0.2204	0.0012	0.018774	0.08791	0.35289

Response	Predictor	estimate	p.value	p.adjust	conf.low	conf.high
6CN	Citrate	0.2425960	0.0151591	0.4699312	0.0472418	0.4379501
6CN	Dimethylamine	0.4450880	0.0326463	0.5060173	0.0370805	0.8530955

Response	Predictor	estimate	p.value	p.adjust	conf.low	conf.high
4HNE	2-Hydroxy isobutyrate	0.6146149	0.0181464	0.5625395	0.1060165	1.1232133
4HNE	Acetate	0.2275781	0.0405842	0.6290545	0.0098668	0.4452894

1.7 **Table S6:** Differences in biomarkers of exposure and effect (median[IQR]) by treatment period (TR=ug/L; adj_TR=ug/g). Biomarkers were adjusted for creatinine levels to account for urine dilution (related to Table 1)

	**Overall**	**Evening**	**Morning**
	**n=187**	**n=88**	**n=99**
6-CN_TR	0.10 [0.03, 0.21]	0.20 [0.09, 0.44]	0.03 [0.03, 0.11]
3-PBA_TR	2.07 [1.14, 4.49]	2.57 [1.44, 4.87]	1.78 [0.97, 3.61]

4-HNE_TR	133.7 [99.3 208.6]	128.5 [94.54, 202.18]	138.7 [110.85, 227]
Cortisol_TR	9.87 [2.80, 22.96]	9.41 [2.65, 19.93]	10.32 [3.13, 24.71]
6-CN_adj_TR	0.06 [0.02, 0.17]	0.16 [0.04, 0.43]	0.04 [0.02, 0.09]
3-PBA_adj_TR	1.70 [0.62, 3.77]	2.31 [0.92, 4.45]	1.29 [0.51, 2.74]
4-HNE_adj_TR	106.6 [72.8, 157.8]	104.7 [74, 165]	106.8 [69.6, 152.3]
Cortisol_adj_TR	6.7 [2.04, 14.80]	6.28 [2.34, 18.82]	6.79 [1.72, 13.41]

2. **Data S1: Food Frequency Questionnaire (FFQ) as validated in Greek (related to STAR Methods)**

<u>ΕΡΩΤΗΜΑΤΟΛΟΓΙΟ ΣΥΧΝΟΤΗΤΑΣ ΔΙΑΤΡΟΦΗΣ</u>

ΟΝΟΜΑΤΕΠΩΝΥΜΟ: ………………………………………………………

ΗΜΕΡΟΜΗΝΙΑ: ………………………………………………………………….

Τον τελευταίο μήνα, πόσες φορές κατανάλωσες τις παρακάτω τροφές: π.χ. αν κατανάλωσες 1 ποτήρι γάλα 1 φορά την ημέρα τότε θα γράψεις 1 στη στήλη <u>φορές/ημέρα</u> και 7 στη στήλη <u>φορές/εβδομάδα</u>, αν είναι 1 φορά την εβδομάδα τότε θα γράψεις 1 στη στήλη <u>φορές/εβδομάδα,</u> αν είναι 1 φορά το μήνα τότε θα γράψεις 1 κάτω από τη στήλη <u>φορές/μήνα</u> κι αν είναι ποτέ ή δεν θυμάσαι/δεν γνωρίζεις τότε θα σημειώσεις √ στο αντίστοιχο κουτάκι (βλέπε πίνακα παρακάτω).

**Τρόφιμα**	**Κατανάλωση**	**Συχνότητα**				
		Ποτέ	Φορές/ ημέρα	Φορές/ εβδομάδα	Φορές/ μήνα	Δεν θυμάμαι/ Δεν γνωρίζω
Γάλα (ποτήρι του νερού, 250ml)	1 ποτήρι/ημέρα		1	7		
	2 ποτήρια/μήνα				2	

Γιαούρτι πλήρες (συσκευασία 200γρ)	3 κεσεδάκια/ εβδομάδα			3		
Κρέας (μερίδα=90γρ)	2 μερίδες/1 ημέρα/ εβδομάδα		2	1		
	2 μερίδες/2 ημέρες/ εβδομάδα		2	2		
	Ποτέ	√				
Ψωμί (φέτα=30γρ)	2 φέτες/ημέρα		2	7		
Τυρί (μερίδα=30γρ)	2 μερίδες/εβδομάδα			2		
Λαχανικά βραστά , π.χ. κουνουπίδι κ.τ.λ. (½ φλιντζάνι)	1 φλιντζάνι/ημέρα		2 (1×2)	7		
	2 φλιτζάνια/ εβδομάδα			4 (2×2)		
Όσπρια (½ φλιτζάνι)	3 φλιτζάνια/μήνα				6 (3×2)	
Φρούτα (αριθμός)	3 φρούτα/ημέρα		3	7		

ΕΡΩΤΗΜΑΤΟΛΟΓΙΟ

**ΤΡΟΦΙΜΑ**	**ΣΥΧΝΟΤΗΤΑ**				
	Ποτέ	Φορές/ ημέρα	Φορές/ εβδομάδ α	Φορές/ μήνα	Δεν θυμάμαι/ Δεν γνωρίζω
1. Γάλα (ένα ποτήρι νερού, 250ml)					
**α.** φρέσκο πλήρες					
**β.** φρέσκο ημιάπαχο					
**γ.** φρέσκο άπαχο					
**δ.** εβαπορέ πλήρες					
**ε.** εβαπορέ ημιάπαχο					
**στ.** εβαπορέ άπαχο					
**ζ.** ζαχαρούχο					
**η.** σοκολατούχο					
2. Γιαούρτι (σκέτο ή με φρούτα) (μικρή συσκευασία 200γρ)					
**α.** πλήρες					

ΤΡΟΦΙΜΑ	ΣΥΧΝΟΤΗΤΑ				
	Ποτέ	Φορές/ ημέρα	Φορές/ εβδομάδ α	Φορές/ μήνα	Δεν θυμάμαι/ Δεν γνωρίζω
**β.** ημιάπαχο					
**γ.** άπαχο					
3. Τυρί (μερίδες μεγέθους σπιρτόκουτου, μερίδα=30γρ)					
**α.** κεφαλοτύρι, γραβιέρα, κασέρι, Cheddar					
**β.** φέτα, χαλούμι, φρέσκα αναρή, Edam					
**γ.** μαλακό τυρί/κρεμώδες (π.χ. Cottage, Philadelphia, La vache qui rit)					
Το τυρί που τρώς είναι χαμηλό σε λιπαρά; (σημείωσε √ στο αντίστοιχο κουτάκι)					
**α**. ποτέ					
**β.** μερικές φορές					
**γ.** συνήθως/πάντα					
**δ.** δεν θυμάμαι/δεν γνωρίζω					
4. Αυγά (αριθμός)					

ΤΡΟΦΙΜΑ	ΣΥΧΝΟΤΗΤΑ				
	Ποτέ	Φορές/ ημέρα	Φορές/ εβδομάδ α	Φορές/ μήνα	Δεν θυμάμαι/ Δεν γνωρίζω
5. Δημητριακά προγεύματος (μέτριο μπώλ)					
**α.** Cornflakes, Rice-Krispies, άλλα χαμηλά σε φυτικές ίνες)					
**β.** All-Bran, Fruit 'n' Fiber, Muesli, άλλα ψηλά σε φυτικές ίνες					
**γ.** σοκολατούχα, ζαχαρούχα, π.χ. Coco-Pops, Frosties					
6. Ψωμί, φραντζόλα, πίτα, φρυγανιά, παξιμάδι (φέτα/slice=30γρ)					
**α.** άσπρο					
**β.** χωριάτικο ψωμί/κουλούρι					
**γ.** μαύρο					
**δ.** πολύσπορο					
7. Δημητριακά (μερίδα= ½ φλιτζάνι)					
**α.** ρύζι					
**β.** πλιγούρι (πουργούρι)					

ΤΡΟΦΙΜΑ	ΣΥΧΝΟΤΗΤΑ				
	Ποτέ	Φορές/ ημέρα	Φορές/ εβδομάδ α	Φορές/ μήνα	Δεν θυμάμαι/ Δεν γνωρίζω
**γ.** τραχανάς					
8. Ζυμαρικά					
**α.** μακαρόνια, κριθαράκι (μερίδα= ½ φλιτζάνι)					
**β.** γεμιστά ζυμαρικά (π.χ. ραβιόλες, τορτελλίνια) (μερίδα= ½ φλιτζάνι)					
**γ.** μακαρόνια φούρνου (ένα μέτριο κομμάτι) (αριθμός)					
9. Πατάτες					
**α.** τηγανιτές (μερίδα=20-25 πατάτες)					
**β.** βραστές (μερίδα=2 μικρές πατάτες - 90γρ)					
**γ**. φούρνου (μερίδα=2 μικρές πατάτες - 90γρ)					
**δ**. πουρέ (μερίδα= ½ φλιτζάνι)					
10. Όσπρια π.χ. φακές, φασόλια (μερίδα=½ φλιτζάνι)					

ΤΡΟΦΙΜΑ	ΣΥΧΝΟΤΗΤΑ				
	Ποτέ	Φορές/ ημέρα	Φορές/ εβδομάδ α	Φορές/ μήνα	Δεν θυμάμαι/ Δεν γνωρίζω
11. Κρέας, προϊόντα κρέατος και ψάρια					
**α.** κρέας ή κιμάς: χοιρινό (π.χ. μπριζόλα, σιεφταλιές, σουβλάκι, χοιρινή σούβλα) (μερίδα=90γρ)					
**β.** κρέας: αρνί/κατσίκι (π.χ. αρνίσια σούβλα, αρνί στο φούρνο, κλέφτικο) (μερίδα=90γρ)					
**γ.** κρέας ή κιμάς: βοδινό (π.χ. στέικ, hamburger) (μερίδα=90γρ)					
**δ.** κοτόπουλο ή κουνέλι (μερίδα=90γρ)					
**ε.** συκώτι (μερίδα=90γρ)					
**στ.** αλλαντικά (μερίδα=1 φέτα) (εκτός αυτών που περιέχονται σε σάντουϊτς)					
**i.** σαλάμι, μπέικον, μουρτατέλα					
**ii.** λούντζα, χαμ					
**ζ.** λουκάνικα (μερίδα=1 μέγεθος φραγκφούρτης) (εκτός αυτών που περιέχονται σε λουκανικόπιτες)					
**η.** κονσέρβες κρέατος (π.χ. ZWAN) (μερίδα=120γρ)					

ΤΡΟΦΙΜΑ	ΣΥΧΝΟΤΗΤΑ				
	Ποτέ	Φορές/ ημέρα	Φορές/ εβδομάδ α	Φορές/ μήνα	Δεν θυμάμαι/ Δεν γνωρίζω
**θ.** κονσέρβες ψαριού (π.χ. τόνος) (μερίδα=120γρ)					
**ι.** ψάρι φρέσκο ή κατεψυγμένο, θαλασσινά (οχταπόδι, καλαμάρι κ.α.) (μερίδα=90γρ)					
12. Λαχανικά και φρούτα					
**α.** λαχανικά ωμά, σαλάτες (μερίδα=1 φλιτζάνι)					
**β.** μαγειρευμένα λαχανικά: χόρτα, φασολάκι, κουνουπίδι κ.τ.λ. (μερίδα=½ φλιτζάνι)					
**γ.** φρέσκα φρούτα (μερίδα= 1 μήλο, 1 μέτριο τσαμπί σταφύλι, 15 κεράσια, 2 χρυσόμηλα, 1 μέτρια φέτα καρπούζι)					
**δ.** χυμοί από φρέσκα φρούτα ή λαχανικά (ποτήρι νερού, 250ml)					
13. Λάδι, αλείμματα, και ελιές (που χρησιμοποιούνται στο μαγείρεμα, προστίθενται την διάρκεια του φαγητού στο τραπέζι, αλείφονται στο ψωμί)					
**α.** ελαιόλαδο (κουταλιές σούπας)					
**β.** σπορέλαιο, ηλιανθέλαιο κ.τ.λ. (κουταλιές σούπας)					
**γ.** μαργαρίνη (κουταλάκια γλυκού)					

ΤΡΟΦΙΜΑ	ΣΥΧΝΟΤΗΤΑ				
	Ποτέ	Φορές/ ημέρα	Φορές/ εβδομάδ α	Φορές/ μήνα	Δεν θυμάμαι/ Δεν γνωρίζω
**δ.** μαργαρίνη χαμηλή σε λιπαρά (π.χ. Flora light/Vitalite) (κουταλάκια γλυκού)					
**ε.** βούτυρο (κουταλάκια γλυκού)					
**στ**.μαγιονέζα (κουταλάκια γλυκού)					
**ζ.** ελιές (αριθμός)					
14. Ζάχαρη, μέλι, μαρμελάδα κ.τ.λ.					
**α.** ζάχαρη (κουταλάκια γλυκού)					
**β.** μέλι (κουταλάκια γλυκού)					
**γ.** μαρμελάδα & γλυκά κουταλιού (κουταλάκια γλυκού)					
**δ.** κομπόστα φρούτων (μερίδα=½ φλιτζάνι)					
15. Σοκολάτες, μπισκότα, παγωτά, γλυκά, κέικ					
**α.** μερέντα π.χ. nutella (κουταλάκια γλυκού)					
**β.** σοκολάτες (π.χ. μια σοκολάτα ΙΟΝ=45γρ) προσδιόρισε					

ΤΡΟΦΙΜΑ	ΣΥΧΝΟΤΗΤΑ				
	Ποτέ	Φορές/ ημέρα	Φορές/ εβδομάδ α	Φορές/ μήνα	Δεν θυμάμαι/ Δεν γνωρίζω
**γ.** γκοφρέτες (π.χ. μια σοκοφρέτα=40γρ)					
**δ.** μπισκότα, κουλουράκια (μερίδα=1 μπισκότο ή κουλουράκι)					
**ε.** παγωτά (μερίδα=1 μπαλάκι/scoop)					
**στ.** κρέμες (π.χ. καραμελέ, μους) (1 μέτριο μπώλ)					
**ζ.** κέικ, τούρτες, τάρτες, γλυκίσματα (1 μέτριο κομμάτι)					
16. Σνακς και γεύματα					
**α**. τυρόπιτες, χαλουμόπιτες, πουρέκια, σουφλέ (αριθμός)					
**β.** λουκανικόπιτες, κοτόπιτες (αριθμός)					
**γ.** ταχινόπιτες, ελιόπιτες (αριθμός)					
**β.** κρουασάν, ντόνατς (αριθμός)					
**δ.** σάντουιτς (ζαμπόν-τυρί, λούντζα-χαλούμι) (αριθμός)					

ΤΡΟΦΙΜΑ	ΣΥΧΝΟΤΗΤΑ				
	Ποτέ	Φορές/ ημέρα	Φορές/ εβδομάδ α	Φορές/ μήνα	Δεν θυμάμαι/ Δεν γνωρίζω
ε. σάντουιτς (ζαμπόν ή λούντζα ή γαλοπούλα) (αριθμός)					
**ε.** σάντουιτς (τυρί ή χαλούμι) (αριθμός)					
**στ**. σάντουϊτς (τόνος κ.α.) (αριθμός)					
**ζ.** πίτσα (ένα κομμάτι από μεγάλου μεγέθους πίτσα) (αριθμός)					
**η**. γεύματα «φαστ φουντ» (π.χ. McDonald's, KFC) (αριθμός)					
**θ.** μουσακάς (ένα μέτριο κομμάτι) (αριθμός)					
**ι.** γεμιστά ή ντολμάδες (αριθμός)					
17. Γαριδάκια, πατατάκια, πόπ κόρν (μικρά σακουλάκια)					
18. Ξηροί καρποί (φλιτζανάκια καφέ)					
19. Ποτά και καφέδες					
**α. Κρασί** (ποτήρια)					
**β. Μπύρα** (ποτήρια)					

ΤΡΟΦΙΜΑ	ΣΥΧΝΟΤΗΤΑ				
	Ποτέ	Φορές/ ημέρα	Φορές/ εβδομάδ α	Φορές/ μήνα	Δεν θυμάμαι/ Δεν γνωρίζω
**γ. Άλλα οινοπβευματώδη** (ουίσκι, βότκα, ζιβανία, κ.α.) (μεζούρες)					
**δ. Νερό** (ποτήρια)					
**ε. Καφές** (κυπριακός, νες-καφέ, φραπέ, καπουτσίνο, κ.α.) (φλιτζάνια)					
**i. με ζάχαρη** (προσδιόρισε την ποσότητα, π.χ. 1 κουταλάκι, ...)					
**ii. με γάλα** (προσδιόρισε την ποσότητα, π.χ. ½ ποτήρι γάλα, ...)					
**στ. αναψυκτικά** (τενεκεδάκια, 330cc)					
**i.** αναψυκτικά με ζάχαρη					
**ii.** αναψυκτικά χωρίς ζάχαρη (diet)					
**ζ. χυμοί** (1 κουτί/1 ποτήρι 250ml)					
**i.** χυμοί συμπυκνωμένοι (π.χ. Λανίτη, ΚΕΑΝ κ.τ.λ.)					

ΤΡΟΦΙΜΑ	ΣΥΧΝΟΤΗΤΑ				
	Ποτέ	Φορές/ ημέρα	Φορές/ εβδομάδ α	Φορές/ μήνα	Δεν θυμάμαι/ Δεν γνωρίζω
**ii.** φρουτοποτά (π.χ. Κεανίτα)					
**δ. άλλα ποτά** (1 κουτί/μπουκάλι)					
**i.** ισοτονικά (π.χ. Lucozade, Gatorade)					
**ii.** energy drinks (π.χ. Redbull, Shark)					
***20.* Συμπληρώματα**					
**α.** βιταμίνες (προσδιόρισε ....)					
**β.** άλλα (π.χ. πρωτεΐνες)<br>(προσδιόρισε ……………………..............)					
***21.* Άλλα φαγητά**					
**α.**					
**β.**					

3. **Data S2: 24-hour Dietary Recall (24-DHR) in Greek language of the study participants (related to STAR Methods)**

Ανάκληση 24ώρου -1η

Ημερομηνία: …………………………..

Ημέρα μελέτης (παραμονή baseline): **………………………**

Ο ερευνητής θα κάνει ανάκληση 24ώρου για τη συλλογή δεδομένων ως προς τη διαιτητική πρόσληψη του συμμετέχοντα για το προηγούμενο 24ωρο. Το ερωτηματολόγιο θα έχει την εξής δομή. Επισυνάπτεται σχετικό έντυπο για τον ερευνητή.

Ώρα κατανάλωσης	Τροφή ή ποτό που καταναλώθηκε (είδος)	Ποσότητα	Τρόπος παρασκευής	Μέρος

4. **Data S3: RedCap® online diary (related to STAR Methods)**

For each participant a unique online registry was created in RedCap®. These questionnaires consisted of 3 pillars: Demographics, Anthropometrics and the Daily questionnaires for the 14 days that was the duration of the study. The demographics were completed by the lead researcher during the first appointment. The anthropometrics were also filled on three different time points by the lead researcher: at baseline (first appointment) and during the two follow-up appointments (end of first week, end of second week respectively). Participants did not have access to these sections of the online questionnaires.

During the first appointment, participants were sent a unique link which provided access to their online diaries in RedCap®.They were asked to complete the diary on a daily basis, and they were asked to complete each day for the previous one. They received Twilio® notifications as a reminder every morning. Each participants had to complete 14 questionnaires (7 for the first week and 7 for the second week). In order for a participant to be able to complete a diary he/she had to complete the previous one, otherwise the system did not allow him/her to proceed. The diaries contained information regarding fruit/vegetable portion compliance and time compliance (i.e. consumption within the indicated time-restricted window), pesticide exposure, sleep duration, smoking habits, physical activity, fluid intake, medication and supplements usage. A demo sample of the RedCap® questionnaire can be found on this link (containing all thee pillars):

https://redcap.cut.ac.cy/surveys/?s=Y8EEPPW7KTEJAFDP

5. CONSORT 2010 checklist for:
A time differentiated dietary intervention effect on the biomarkers of exposure to pyrethroids and neonicotinoids pesticides.

Section/Topic	Item No	Checklist item	Reported on page No
**Title and abstract**			
	1a	Identification as a randomised trial in the title	1
	1b	Structured summary of trial design, methods, results, and conclusions (for specific guidance see CONSORT for abstracts)	2
**Introduction**			
Background and objectives	2a	Scientific background and explanation of rationale	2-3
	2b	Specific objectives or hypotheses	3
**Methods**			
Trial design	3a	Description of trial design (such as parallel, factorial) including allocation ratio	6, 12
	3b	Important changes to methods after trial commencement (such as eligibility criteria), with reasons	n/a
Participants	4a	Eligibility criteria for participants	6
	4b	Settings and locations where the data were collected	6
Interventions	5	The interventions for each group with sufficient details to allow replication, including how and when they were actually administered	6-7
Outcomes	6a	Completely defined pre-specified primary and secondary outcome measures, including how and when they were assessed	8
	6b	Any changes to trial outcomes after the trial commenced, with reasons	n/a
Sample size	7a	How sample size was determined	n/a
	7b	When applicable, explanation of any interim analyses and stopping guidelines	n/a

Randomisation:			
Sequence generation	8a	Method used to generate the random allocation sequence	6
	8b	Type of randomisation; details of any restriction (such as blocking and block size)	6
Allocation concealment mechanism	9	Mechanism used to implement the random allocation sequence (such as sequentially numbered containers), describing any steps taken to conceal the sequence until interventions were assigned	6
Implementation	10	Who generated the random allocation sequence, who enrolled participants, and who assigned participants to interventions	6
Blinding	11a	If done, who was blinded after assignment to interventions (for example, participants, care providers, those assessing outcomes) and how	6
	11b	If relevant, description of the similarity of interventions	n/a
Statistical methods	12a	Statistical methods used to compare groups for primary and secondary outcomes	8-9
	12b	Methods for additional analyses, such as subgroup analyses and adjusted analyses	8-9
**Results**			
Participant flow (a diagram is strongly recommended)	13a	For each group, the numbers of participants who were randomly assigned, received intended treatment, and were analysed for the primary outcome	SI.p3
	13b	For each group, losses and exclusions after randomisation, together with reasons	3
Recruitment	14a	Dates defining the periods of recruitment and follow-up	3
	14b	Why the trial ended or was stopped	n/a
Baseline data	15	A table showing baseline demographic and clinical characteristics for each group	14-16
Numbers analysed	16	For each group, number of participants (denominator) included in each analysis and whether the analysis was by original assigned groups	14-16

Outcomes and estimation	17a	For each primary and secondary outcome, results for each group, and the estimated effect size and its precision (such as 95% confidence interval)	17
	17b	For binary outcomes, presentation of both absolute and relative effect sizes is recommended	n/a
Ancillary analyses	18	Results of any other analyses performed, including subgroup analyses and adjusted analyses, distinguishing pre-specified from exploratory	n/a
Harms	19	All important harms or unintended effects in each group (for specific guidance see CONSORT for harms)	n/a
**Discussion**			
Limitations	20	Trial limitations, addressing sources of potential bias, imprecision, and, if relevant, multiplicity of analyses	5
Generalisability	21	Generalisability (external validity, applicability) of the trial findings	n/a
Interpretation	22	Interpretation consistent with results, balancing benefits and harms, and considering other relevant evidence	4-5
**Other information**			
Registration	23	Registration number and name of trial registry	6
Protocol	24	Where the full trial protocol can be accessed, if available	6
Funding	25	Sources of funding and other support (such as supply of drugs), role of funders	9

6. Data S4: NMR Methodology (related to STAR Methods)

1H-NMR metabolomic profiling

Urine samples were profiled by ^{1}H-NMR which allowed the quantification of 33 metabolites in urine in raw concentration (uM) from NMR tube (including creatinine to account for urine dilution effects).

Material and methods

Disodium hydrogen phosphate, sodium hydrogen phosphate and 3-(trimethylsilyl) propionic-2,2,3,3-d^4 acid sodium salt (TSP) were purchased from Sigma Aldrich. Deuterated water 99.8% from Deutero. Extra pure water from Milli-Q water system, Millipore.

Sample preparation

The methodology followed for the extraction of urine metabolites was proton nuclear magnetic resonance (1H-NMR). The urine sample was mixed (1:1, v/v) with phosphate buffered saline containing with 3-(trimethylsilyl)propionic-2,2,3,3-d4 acid sodium salt (TSP) (Sigma Aldrich) and placed on a 5nm NMR tube for direct analysis by 1H-NMR.

1HNMR spectra were recorded at 300K on an Avance III 600 spectrometer (Bruker®, Karlsruhe, Germany) operating at a proton frequency of 600.20MHz using a 5mm PBBO gradient probe. The sample preparation procedure was done with the Gilson 215 liquid handler for improve reproducibility as published.[1] Urine vials of 300µL kept at 4°C into Peltier racks were added to 96 NMR deep well plate and then 300 µL of 0.25M PBS buffer in D_2O were added (pH 7.4, 25 °C, TSP 0.4mM). The mixture was vortexed until complete homogenization and centrifuged (around 800 g, 5min, 4 °C) to precipitate particulates. The upper clear solution was transferred into a 5mm o.d. NMR tube. Sample jet 96-racks of NMR tubes were kept at 4°C during NMR acquisition.

NMR analysis

Diluted urine aqueous samples were measured and recorded in procno 11 using a one-dimensional ^{1}H pulse experiments were carried out using the nuclear overhauser effect spectroscopy (NOESY). Presaturation sequence (RD–90°–t1–90°–tm–90° ACQ) to suppress the residual water peak, and the mixing time was set at 100 ms. Solvent presaturation with irradiation power of 150 µW was applied during recycling delay (RD = 5s) and mixing time (noesypr1d pulse program in Bruker library) to eliminate the residual water. The 90° pulse length was calibrated for each sample and varied from 11.05 to 12.38 ms. The spectral width was 9.6 kHz (16 ppm), and a total of 256 transients were collected into 64k data points for each ^{1}H spectrum. The exponential line broadening applied before Fourier transformation was of 0.3 Hz.

The frequency domain spectra were phased, baseline-corrected and referenced to TSP signal (d= 0 ppm) using TopSpin software (version 3.6, Bruker). TSP was used for chemical shift reference to 0 ppm. For quantification, the Eretic Signal was used that was calibrated with 2mM sucrose reference sample to a 1.0807 mM value (10 October, 2021).

All acquired ^{1}H NMR were compared to references of the pure selected compounds with the metabolic profiling AMIX spectra database (Bruker), HMDB, and Chenomx and built in-house databases for metabolite identification. In addition, we assigned metabolites by ^{1}H–^{1}H homonuclear correlation (COSY and TOCSY) and ^{1}H–^{13}C heteronuclear (HSQC) 2D NMR experiments and by correlation with pure compounds run in-house, when needed. After pre-processing, specific ^{1}H NMR regions identified in the spectra were integrated using the MATLAB software package. Curated identified regions across the spectra that were integrated using the same software package were exported to excel spreadsheet to evaluate robustness of the different ^{1}H NMR signals and to calculate concentrations. Quality controls measured in each 96-plate batch were also integrated. After NMR raw dataset curation and removal of water and reference signals, spectra were aligned, normalized and detected metabolites were quantified using the profiling process [2]

References

1. Herrero P, Rodríguez MA, Ras MR, del Pino A, Arola L, Canela N. Metabolomics analyses to investigate the role of diet and physical training. In: D'Alessandro A, ed. Methods in Molecular Biology. Vol 1978. Humana Press Inc.; 2019:403-430.

2. Vinaixa M, Rodriguez MA, Rull A, et al., 2010. Metabolomic Assessment of the Effect of Dietary Cholesterol in the Progressive Development of Fatty Liver Disease. J Proteome Res. 9(5):2527-2538.
