# Supplementary material for: A time differentiated dietary intervention effect on the biomarkers of exposure to pyrethroids and neonicotinoids pesticides
Source: iScience. 2022 Dec 22;26(2):105847. doi: 10.1016/j.isci.2022.105847 (PMC9874006; doi:10.1016/j.isci.2022.105847)
Supplement: Data S5. Sensitivity analysis, related to Figure 2 [file mmc2.pdf]

Data S5: Sensitivity analysis of the mixed effect models (related to Table 2-3)
Excluding participants with compliance <80% in either one of the 2 treatments

6-CN model

	cn adj TR log S		
Predictors	Estimates	CI	p
(Intercept)	-0.37	-1.37 – 0.63	0.463
phase [Morning]	-1.81	-2.56 – -1.05	<0.001
cn adj BL log S	0.09	-0.09 – 0.26	0.344
days	-0.01	-0.12 – 0.10	0.902
sex [Male]	-0.66	-1.21 – -0.11	0.019
age	0.03	0.01 – 0.05	0.002
phase [Morning] * days	0.09	-0.06 – 0.25	0.243
Random Effects			
σ2	1.32		
τ00 record_id	0.17		
ICC	0.11		
N record_id	33		
Observations	198		
Marginal R2 / Conditional R	0.341 / 0.415		

3-PBA model

	pb adj TR log S		
Predictors	Estimates	CI	p
(Intercept)	0.11	-0.97 – 1.18	0.846
phase [Morning]	-0.98	-1.60 – -0.37	0.002
pb adj BL log S	0.07	-0.11 – 0.25	0.438
days	-0.10	-0.19 – -0.01	0.034
sex [Male]	-1.02	-1.64 – -0.40	0.001
age	0.02	-0.00 – 0.05	0.072
phase [Morning] * days	0.09	-0.03 – 0.22	0.151
Random Effects			
σ2	0.88		
τ00 record_id	0.34		
ICC	0.28		
N record_id	33		
Observations	198		
Marginal R2 / Conditional R	0.269 / 0.473		

4-HNE model

	hne adj TR log S		
Predictors	Estimates	CI	p
(Intercept)	-0.77	-1.31 – -0.22	0.006
phase [Morning]	0.12	-0.27 – 0.52	0.534
hne adj BL log S	0.12	-0.01 – 0.26	0.079
days	0.03	-0.03 – 0.09	0.307
sex [Male]	-0.28	-0.57 – 0.01	0.061

age	0.02	0.01 – 0.03	0.001
phase [Morning] * days	-0.04	-0.12 – 0.04	0.370
Random Effects			
σ2	0.36		
τ00 record_id	0.06		
ICC	0.14		
N record_id	33		
Observations	197		
Marginal R2 / Conditional R:	0.182 / 0.294		

4-HNE ~ 3-PBA model

	hne adj TR log S		
Predictors	Estimates	CI	p
(Intercept)	-0.62	-1.05 – -0.19	0.005
hne adj BL log S	0.09	-0.03 – 0.22	0.127
pb adj TR log S	0.22	0.14 – 0.29	<0.001
days	0.02	-0.02 – 0.06	0.235
sex [Male]	-0.05	-0.31 – 0.20	0.680
age	0.01	0.00 – 0.02	0.006
Random Effects			
σ2	0.32		
τ00 record_id	0.03		
ICC	0.08		
N record_id	33		
Observations	197		
Marginal R2 / Conditional R:	0.306 / 0.362		

4-HNE ~ 6-CN model

	hne adj TR log S		
Predictors	Estimates	CI	p
(Intercept)	-0.57	-1.06 – -0.09	0.021
hne adj BL log S	0.11	-0.02 – 0.24	0.107
cn adj TR log S	0.11	0.05 – 0.17	0.001
days	0.01	-0.03 – 0.05	0.720
sex [Male]	-0.21	-0.49 – 0.07	0.147
age	0.02	0.00 – 0.03	0.006
Random Effects			
σ2	0.34		
τ00 record_id	0.05		
ICC	0.12		
N record_id	33		
Observations	197		
Marginal R2 / Conditional R:	0.228 / 0.322		
