# Supplementary material for: A time differentiated dietary intervention effect on the biomarkers of exposure to pyrethroids and neonicotinoids pesticides
Source: iScience. 2022 Dec 22;26(2):105847. doi: 10.1016/j.isci.2022.105847 (PMC9874006; doi:10.1016/j.isci.2022.105847)
Supplement: Data S6. Descriptives, related to Table 1 [file mmc3.pdf]

Data S6: Additional Descriptive statistical analysis of the participant characteristics by study group (related to STAR Methods)

FFQ descriptives stratified by Groups A and B

	Overall	A	B	p	test
n	38	21	17		
fruits_per_day (mean (SD))	1.98 (1.29)	2.05 (1.40)	1.88 (1.18)	0.690	
veg_per_day (mean (SD))	2.60 (2.30)	2.43 (1.88)	2.81 (2.79)	0.626	
fruits_per_week (mean (SD))	13.89 (9.24)	14.48 (10.13)	13.18 (8.26)	0.672	
veg_per_week (mean (SD))	18.20 (16.13)	17.03 (13.16)	19.64 (19.52)	0.626	
total_daily_fv (mean (SD))	4.58 (3.10)	4.49 (2.91)	4.69 (3.40)	0.845	
total_weekly_fv (mean (SD))	32.09 (21.84)	31.51 (20.67)	32.82 (23.83)	0.857	
kcal_bl (mean (SD))	1880.76 (973.34)	2080.81 (1171.46)	1633.63 (598.36)	0.162	
kcal_1st (mean (SD))	1532.89 (557.53)	1565.10 (567.57)	1489.95 (560.58)	0.699	
kcal_2nd (mean (SD))	1663.44 (526.18)	1724.89 (525.34)	1587.54 (533.11)	0.431	

Calories

Calories descriptives

	n	mean	sd	median	se	min	max	range
kcal_bl	38	1880.76	973.34	1637.90	157.90	573.2	6177.4	5604.2

kcal_1st	35	1532.89	557.53	1379.50	94.24	768.0	3367.8	2599.8
kcal_2nd	38	1663.44	526.18	1671.05	85.36	869.5	2740.5	1871.0

Calories per phase

	Overall	Baseline	Evening	Morning	p	test
n	105	35	35	35		
kcal (mean (SD))	1711.45 (734.68)	1920.74 (989.38)	1625.05 (552.99)	1588.55 (545.92)	0.116	

Calories per group

	Overall	A	B	p	test
n	105	60	45		
kcal (mean (SD))	1711.45 (734.68)	1795.43 (843.24)	1599.48 (547.62)	0.177	

Calories percentage from fruits and vegetables per phase

	Overall	Evening	Morning	p	test
n	70	15	20		
fruit_veg_prop (mean (SD))	28.46 (10.86)	29.13 (11.26)	26.15 (8.83)	0.386	

Calories percentage from fruits and vegetables per group

	Overall	A	B	p	test
n	70	40	30		
fruit_veg_prop (mean (SD))	28.46 (10.86)	28.15 (10.33)	28.87 (11.70)	0.787	

Calories difference per phase

	Evening	Morning	p	test
n	35	35		
kcal_difference (mean (SD))	7.00 (661.99)	-247.03 (851.27)	0.190	

Measurements descriptives.

	Overall	Baseline	Evening	Morning	p	test
n	113	38	37	38		
weight (mean (SD))	67.52 (11.78)	67.70 (12.09)	67.52 (11.90)	67.33 (11.68)	0.991	

	Overall	Baseline	Evening	Morning	p	test
n	112	38	36	38		
bmi (mean (SD))	24.76 (2.98)	24.85 (3.07)	24.69 (2.96)	24.74 (2.99)	0.972	

	Overall	Baseline	Evening	Morning	p	test
n	110	38	35	37		
waist (mean (SD))	82.41 (10.41)	82.92 (10.53)	82.65 (10.85)	81.65 (10.12)	0.861	

Measurements differences

	Evening	Morning	p	test
n	37	37		
weight (mean (SD))	-0.18 (1.11)	-0.16 (2.30)	0.959	

	Evening	Morning	p	test
n	36	36		
bmi (mean (SD))	-0.04 (0.37)	-0.03 (0.29)	0.884	

	Evening	Morning	p	test
n	34	34		
waist (mean (SD))	0.29 (2.31)	-0.42 (1.91)	0.181	

Number of physical exercises per group for each day

	Overall	A	B	p	test
n	532	294	238		
num_of_exercises (mean (SD))	0.45 (0.66)	0.51 (0.75)	0.38 (0.52)	0.025	

Number of exercises per phase for each day

	Overall	Evening	Morning	p	test

n	532	266	266		
num_of_exercises (mean (SD))	0.45 (0.66)	0.40 (0.64)	0.50 (0.68)	0.076	

Medicine usage through study.

	Overall
n	38
medicine (median [IQR])	0.00 [0.00, 2.00]
med_prop (median [IQR])	0.00 [0.00, 0.14]

Medicine usage through study only for urine sample days.

	Overall
n	38
medicine (median [IQR])	0.00 [0.00, 1.00]
med_prop (median [IQR])	0.00 [0.00, 0.12]

Sleep duration for all days per week

	Overall	1	2	p	test
n	532	266	266		
duration2 (mean (SD))	7.73 (1.26)	7.67 (1.16)	7.79 (1.36)	0.228	

Sleep duration for all days per phase

	Overall	Evening	Morning	p	test
n	532	266	266		
duration2 (mean (SD))	7.73 (1.26)	7.75 (1.27)	7.72 (1.26)	0.784	

Sleep duration for all days per group

	Overall	A	B	p	test
n	532	294	238		

duration2 (mean (SD))	7.73 (1.26)	7.62 (1.22)	7.87 (1.30)	0.027

Descriptives for mean sleep duration for each participant

	n	mean	sd	median	se	min	max	range
Mean sleep duration	38	7.73	0.68	7.67	0.11	6.36	9.14	2.78

Sleep duration for urine sample days per week

	Overall	1	2	p	test
n	304	152	152		
duration2 (mean (SD))	7.72 (1.27)	7.75 (1.18)	7.69 (1.35)	0.676	

Sleep duration for urine sample days per phase

	Overall	Evening	Morning	p	test
n	304	152	152		
duration2 (mean (SD))	7.72 (1.27)	7.73 (1.24)	7.71 (1.30)	0.924	

Sleep duration for urine sample days per group

	Overall	A	B	p	test
n	304	168	136		
duration2 (mean (SD))	7.72 (1.27)	7.58 (1.21)	7.89 (1.33)	0.038	

Sleep duration for each urine sample day

	Overall	1	3	5	7	8	10	12	14	p	test
n	304	38	38	38	38	38	38	38	38		
duration2 (mean (SD))	7.72 (1.27)	7.74 (1.01)	7.85 (1.22)	7.85 (1.29)	7.55 (1.19)	7.94 (1.47)	7.66 (1.20)	7.64 (1.44)	7.53 (1.31)	0.811	

Liquid consumption per phase,week and group

	Overall	Evening	Morning	p	test
n	532	266	266		

water2 (mean (SD))	1586.70 (744.74)	1580.83 (751.68)	1592.58 (739.12)	0.854	
tea2 (mean (SD))	89.99 (197.63)	76.60 (167.02)	103.38 (223.61)	0.137	
soft_drink2 (mean (SD))	28.90 (94.04)	33.36 (95.31)	24.44 (92.73)	0.287	
coffee2 (mean (SD))	191.14 (219.58)	182.80 (218.18)	199.47 (221.07)	0.382	
alcohol2 (mean (SD))	0.54 (1.24)	0.54 (1.22)	0.55 (1.26)	0.918	
total (mean (SD))	1897.27 (847.68)	1874.13 (832.38)	1920.42 (863.66)	0.525	

	**Overall**	**1**	**2**	**p**	**test**
n	532	266	266		
water2 (mean (SD))	1586.70 (744.74)	1662.59 (749.21)	1510.81 (733.83)	<0.001	
tea2 (mean (SD))	89.99 (197.63)	96.33 (210.15)	83.65 (184.43)	0.302	
soft_drink2 (mean (SD))	28.90 (94.04)	31.48 (101.91)	26.32 (85.58)	0.480	
coffee2 (mean (SD))	191.14 (219.58)	198.10 (239.79)	184.17 (197.53)	0.281	
alcohol2 (mean (SD))	0.54 (1.24)	0.52 (1.24)	0.57 (1.25)	0.522	
total (mean (SD))	1897.27 (847.68)	1989.03 (840.76)	1805.52 (846.20)	<0.001	

	**Overall**	**A**	**B**	**p**	**test**
n	532	294	238		

water2 (mean (SD))	1586.70 (744.74)	1630.10 (751.33)	1533.09 (734.56)	0.135	
tea2 (mean (SD))	89.99 (197.63)	133.50 (227.04)	36.24 (136.11)	<0.001	
soft_drink 2 (mean (SD))	28.90 (94.04)	32.31 (101.28)	24.68 (84.27)	0.353	
coffee2 (mean (SD))	191.14 (219.58)	155.95 (197.12)	234.60 (237.82)	<0.001	
alcohol2 (mean (SD))	0.54 (1.24)	0.61 (1.30)	0.46 (1.16)	0.154	
total (mean (SD))	1897.27 (847.68)	1952.48 (862.81)	1829.07 (825.31)	0.095	

Liquid consumption for urine sample days per phase,week and group

	**Overall**	**Evening**	**Morning**	**p**	**test**
n	304	152	152		
water2 (mean (SD))	1585.94 (739.66)	1572.37 (739.23)	1599.51 (742.28)	0.759	
tea2 (mean (SD))	83.47 (189.65)	69.90 (158.07)	97.04 (216.36)	0.233	
soft_drink 2 (mean (SD))	26.73 (79.67)	31.25 (88.97)	22.20 (69.15)	0.342	
coffee2 (mean (SD))	195.28 (222.02)	192.96 (227.70)	197.60 (216.91)	0.853	
alcohol2 (mean (SD))	0.57 (1.23)	0.62 (1.30)	0.51 (1.15)	0.434	
total (mean (SD))	1891.98 (846.36)	1867.11 (828.24)	1916.86 (866.12)	0.623	

	**Overall**	**1**	**2**	**p**	**test**
n	304	152	152		

water2 (mean (SD))	1585.94 (739.66)	1661.18 (749.67)	1510.69 (724.16)	<0.001	
tea2 (mean (SD))	83.47 (189.65)	90.46 (203.55)	76.48 (175.04)	0.365	
soft_drink2 (mean (SD))	26.73 (79.67)	27.14 (82.54)	26.32 (76.98)	0.907	
coffee2 (mean (SD))	195.28 (222.02)	212.89 (248.22)	177.66 (191.50)	0.035	
alcohol2 (mean (SD))	0.57 (1.23)	0.45 (1.03)	0.68 (1.39)	0.035	
total (mean (SD))	1891.98 (846.36)	1992.13 (851.83)	1791.84 (831.61)	<0.001	

	Overall	A	B	p	test
n	304	168	136		
water2 (mean (SD))	1585.94 (739.66)	1635.42 (752.54)	1524.82 (721.50)	0.195	
tea2 (mean (SD))	83.47 (189.65)	121.28 (216.04)	36.76 (137.87)	<0.001	
soft_drink2 (mean (SD))	26.73 (79.67)	29.02 (78.55)	23.90 (81.24)	0.578	
coffee2 (mean (SD))	195.28 (222.02)	167.20 (202.99)	229.96 (239.72)	0.014	
alcohol2 (mean (SD))	0.57 (1.23)	0.60 (1.19)	0.54 (1.28)	0.680	
total (mean (SD))	1891.98 (846.36)	1953.51 (856.56)	1815.98 (830.42)	0.159	

Liquid consumption descriptives.

	n	mean	sd	median	min	max
water	38	1586	675	1446	633	3000
tea	38	89.9	153	0.0000000	0.0000	500

soft_drink	38	28.9		49	0.0000000		0.0000		178
coffee	38	191.3		171		141	0.0000		617
alcohol	38	0.54	0.721114		0.1785714		0.0000		2.35
total	38	1897		760		1732		661	3566

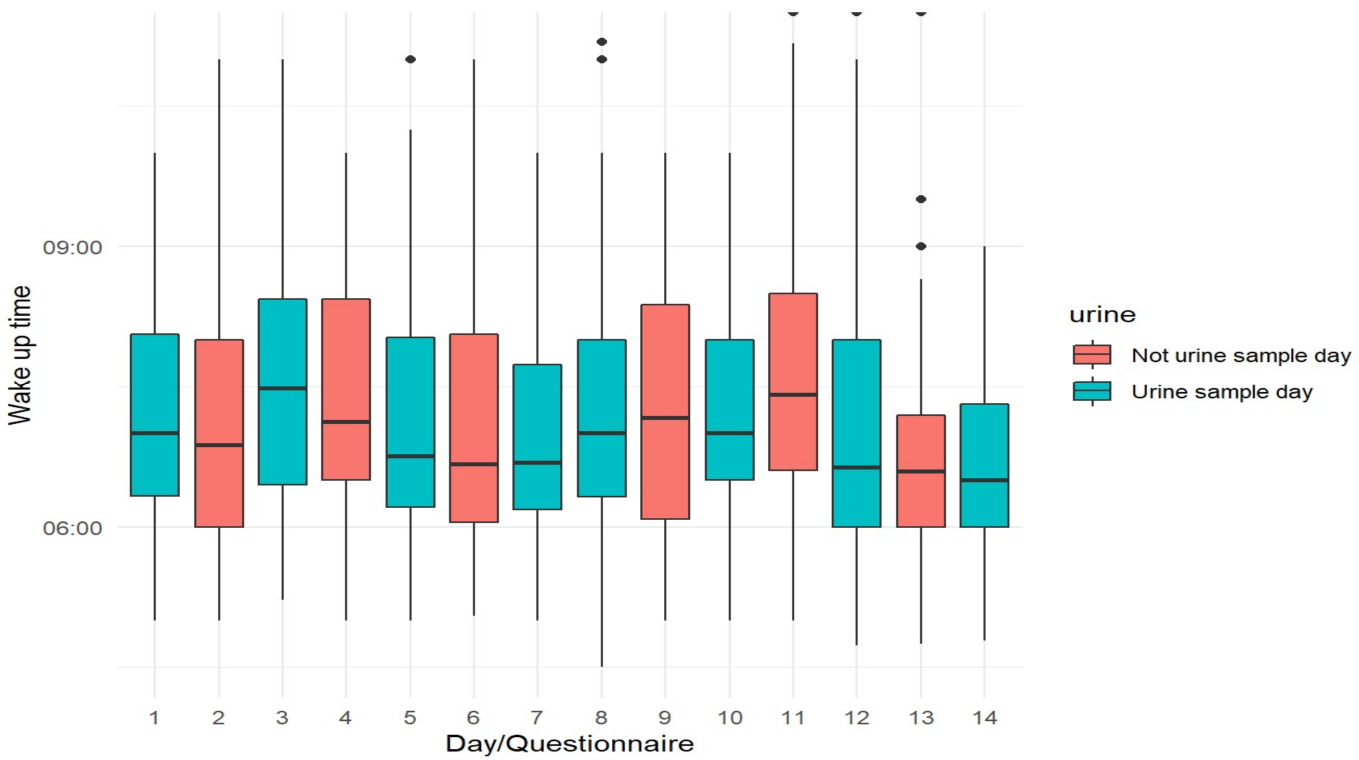

Boxplot of wake up time per phase

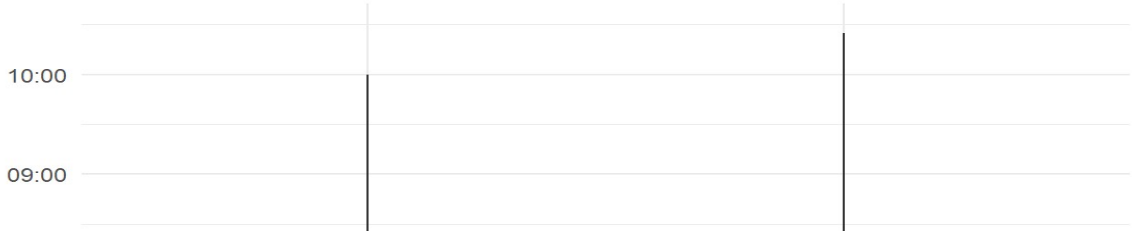

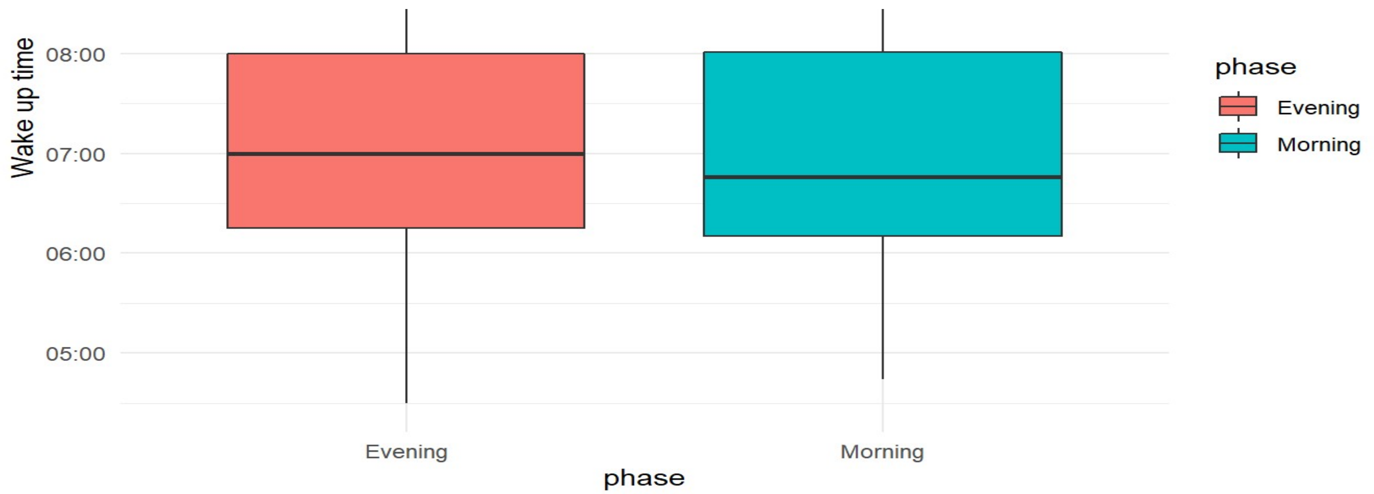
Wake up time
08:00
07:00
06:00
05:00
Evening
Morning
phase
phase
Evening
Morning

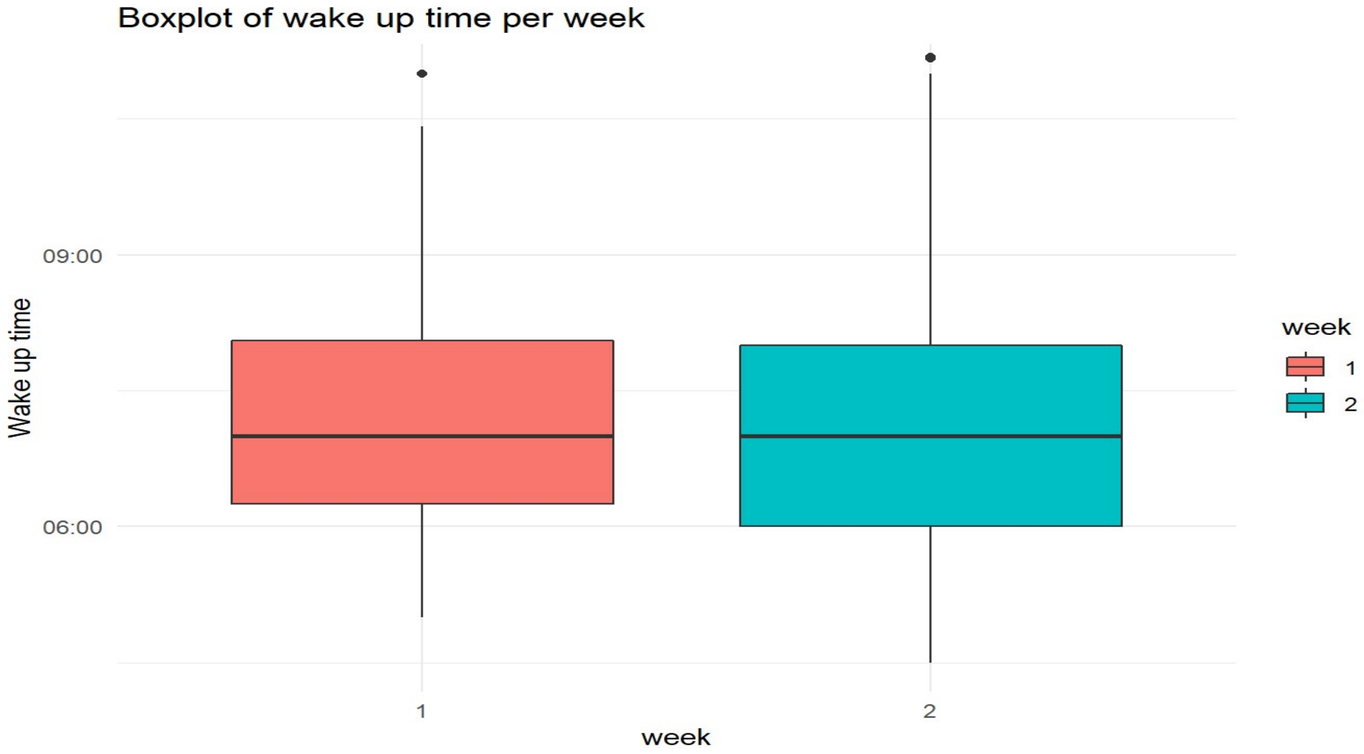
Boxplot of wake up time per week
Wake up time
09:00
06:00
1
2
week
week
1
2

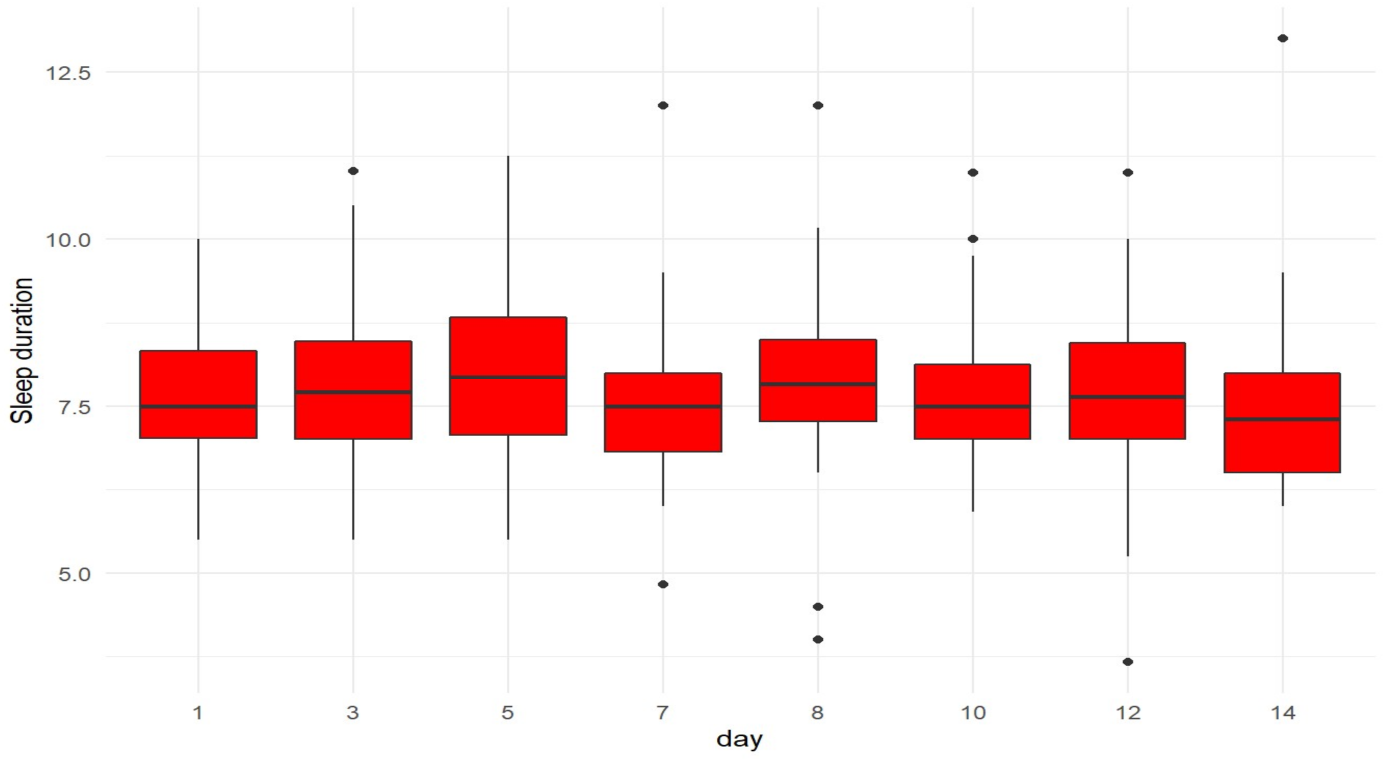

12.5
10.0
7.5
5.0
Sleep duration
1
3
5
7
8
10
12
14
day
