# Supplementary material for: A time differentiated dietary intervention effect on the biomarkers of exposure to pyrethroids and neonicotinoids pesticides
Source: iScience. 2022 Dec 22;26(2):105847. doi: 10.1016/j.isci.2022.105847 (PMC9874006; doi:10.1016/j.isci.2022.105847)
Supplement: Data S7. NMR metabolomics, related to Figure 2 [file mmc4.pdf]

Table S5 (Related to Figure 2): Quantiles of the NMR-based metabolites (quantified, μM), (overall and by phase period).

μM	Phase	mean	sd	min	q25	q50	q75	q90	q95	max
2-Hydroxy isobutyrate	Evening	51,403	32,011	7,500	27,298	43,075	66,079	89,787	114,347	170,260
2-Hydroxy isobutyrate	Morning	56,873	41,135	11,107	30,068	44,942	67,941	104,646	126,929	254,178
2-Hydroxy isobutyrate	Overall	54,154	36,930	7,500	28,502	44,149	67,646	97,785	122,610	254,178
2-Mehtyl-3-Keto valeric acid	Evening	123,417	76,415	13,431	70,265	107,531	168,213	225,762	294,229	365,882
2-Mehtyl-3-Keto valeric acid	Morning	134,656	88,224	14,192	69,925	113,927	174,358	262,630	335,643	449,120
2-Mehtyl-3-Keto valeric acid	Overall	129,070	82,634	13,431	69,951	111,020	169,295	247,319	304,023	449,120
3-Hydroxy isovaleric acid	Evening	83,717	63,793	13,630	44,757	69,308	103,758	155,193	190,442	616,761
3-Hydroxy isovaleric acid	Morning	87,538	62,243	10,332	45,992	70,038	107,527	173,681	218,889	341,475
3-Hydroxy isovaleric acid	Overall	85,639	62,952	10,332	45,811	69,560	106,644	167,302	200,263	616,761
3-Methyl-2-oxo valeric acid	Evening	134,396	85,056	12,351	75,077	114,103	178,017	240,514	319,068	482,056
3-Methyl-2-oxo valeric acid	Morning	150,186	101,362	19,003	71,158	126,548	197,335	278,549	324,727	656,947
3-Methyl-2-oxo valeric acid	Overall	142,339	93,807	12,351	72,716	120,270	189,190	272,766	325,435	656,947
Acetate	Evening	215,832	406,328	14,300	90,915	146,633	205,057	304,042	400,700	4,419,906
Acetate	Morning	208,531	219,797	32,898	94,615	151,950	236,484	367,663	492,786	2,013,068
Acetate	Overall	212,160	325,645	14,300	92,046	150,143	215,400	340,921	444,089	4,419,906
Alanine	Evening	304,291	200,837	36,546	154,099	249,949	412,283	620,822	695,179	1,028,343
Alanine	Morning	332,355	217,707	38,286	178,829	274,961	433,909	664,493	773,637	1,054,973
Alanine	Overall	318,407	209,648	36,546	165,854	267,034	418,241	641,801	728,167	1,054,973
Betaine	Evening	55,140	35,363	6,275	28,249	47,375	76,492	104,709	125,883	183,376
Betaine	Morning	56,653	35,599	6,709	28,257	47,865	80,031	104,830	128,378	185,408
Betaine	Overall	55,901	35,436	6,275	28,243	47,862	77,230	104,904	126,900	185,408
Choline	Evening	53,998	40,073	3,730	24,338	40,677	72,354	109,338	125,412	247,894

Choline	Morning	59,433	45,773	5,371	25,059	46,795	76,802	122,612	143,412	252,906
Choline	Overall	56,732	43,056	3,730	24,518	45,188	76,222	113,562	140,141	252,906
cis-Aconitate	Evening	697,228	529,488	50,487	384,719	543,708	853,068	1,241,713	1,786,715	3629.67
cis-Aconitate	Morning	598.08	407.97	77,053	328.44	508.27	810,172	1001.09	1229.54	3148.96
cis-Aconitate	Overall	647,357	474,179	50,487	352,962	528,263	815,646	1,123,358	1,340,320	3,629,675
Citrate	Evening	2,451,092	1,448,639	414,126	1,529,488	2,034,576	3,040,702	4,410,010	5,439,492	7,146,868
Citrate	Morning	2,482,648	1,542,727	276,069	1,353,897	2,144,870	3,177,198	4,640,253	6,071,216	7,448,128
Citrate	Overall	2,466,965	1,494,529	276,069	1,416,747	2,103,691	3,101,139	4,540,380	5,840,865	7,448,128
Creatine/creatine phosphate	Evening	714,993	886,788	47,016	242,299	409,424	784,923	1,418,236	2,710,483	6,031,539
Creatine/creatine phosphate	Morning	854,975	1,136,086	46,902	252,113	489,512	873,108	1,645,497	3,171,513	7,383,255
Creatine/creatine phosphate	Overall	785,406	1,020,707	46,902	247,084	463,949	854,934	1,604,153	2,863,402	7,383,255
Creatinine	Evening	7,748,461	4,759,281	1,226,387	4,100,902	6,597,189	########	########	########	23,332,191
Creatinine	Morning	8,414,341	5,033,605	1,414,730	4,533,401	7,289,679	########	########	########	28,902,475
Creatinine	Overall	8,083,406	4,903,139	1,226,387	4,287,209	6,927,067	########	########	########	28,902,475
Dimethyl sulfone	Evening	46,810	28,864	3,921	25,323	39,393	63,264	81,821	109,902	153,131
Dimethyl sulfone	Morning	49,534	29,337	6,327	28,607	42,746	64,523	90,384	107,278	154,891
Dimethyl sulfone	Overall	48,180	29,091	3,921	26,653	40,211	63,789	86,063	109,099	154,891
Dimethylamine	Evening	541,973	368,142	66,796	264,868	450,021	687,710	963,128	1,430,566	1,966,604
Dimethylamine	Morning	550,507	342,073	85,633	319,069	502,600	656,632	954,459	1,303,545	2,021,190
Dimethylamine	Overall	546,265	354,757	66,796	292,311	474,921	675,359	964,801	1,324,215	2,021,190
Formate	Evening	132,316	122,886	3,366	62,818	110,279	168,856	227,102	283,409	1,335,833
Formate	Morning	126,610	82,700	14,856	75,161	109,708	160,597	239,137	263,478	560,691
Formate	Overall	129,445	104,500	3,366	67,903	109,993	165,477	236,843	271,195	1,335,833
Fumarate	Evening	3,235	3,345	0.000	0.466	2,391	4,464	7,511	9,418	17,106
Fumarate	Morning	3,829	3,727	0.006	0.678	3,017	5,175	8,959	11,682	17,263
Fumarate	Overall	3,534	3,549	0.000	0.542	2,900	4,822	8,182	11,126	17,263
Glycine	Evening	990,499	667,308	161,155	544,991	802,733	1,274,672	1,772,591	2,047,347	4,051,590
Glycine	Morning	1,122,248	907,861	116,089	622,352	804,234	1,341,250	2,035,918	3,357,680	5,350,487
Glycine	Overall	1,056,770	798,959	116,089	571,309	803,484	1,311,212	1,862,480	2,744,885	5,350,487
Hippurate	Evening	2,067,744	1,588,094	137,264	975,183	1,632,478	2,593,645	4,133,034	5,207,154	10,790,793
Hippurate	Morning	2,065,647	1,548,167	272,808	1,046,422	1,652,478	2,454,990	3,998,918	5,079,128	9,195,923

Hippurate	Overall	2,066,689	1,565,766	137,264	981,319	1,642,478	2,534,284	4,156,363	5,243,175	10,790,793
Indoxyl Sulfate	Evening	252,806	153,264	22,755	127,296	226,488	349,144	462,918	523,582	771,168
Indoxyl Sulfate	Morning	285,813	263,367	47,294	142,067	220,563	349,610	494,536	635,184	2,528,861
Indoxyl Sulfate	Overall	269,409	216,096	22,755	137,355	224,661	349,446	478,214	576,250	2,528,861
Lactate	Evening	331,032	234,699	63,096	175,705	274,811	434,913	625,345	723,759	1,961,300
Lactate	Morning	357,222	236,412	60,122	193,502	291,091	441,717	657,447	879,001	1,193,532
Lactate	Overall	344,206	235,571	60,122	182,020	285,579	438,111	647,333	840,424	1,961,300
Malonate	Evening	334,366	230,907	40,786	167,412	262,348	440,375	724,483	777,658	1,172,751
Malonate	Morning	337,795	225,416	38,967	155,351	282,092	486,328	653,724	768,456	1,086,868
Malonate	Overall	336,091	227,823	38,967	158,834	267,609	478,699	675,364	777,398	1,172,751
N-methyl-2-pyridone-5-carboxamide (2-PY)	Evening	100,784	73,731	0.005	49,552	93,152	139,732	194,344	252,864	339,397
N-methyl-2-pyridone-5-carboxamide (2-PY)	Morning	114,825	92,145	0.016	53,739	103,260	154,080	217,504	280,991	574,801
N-methyl-2-pyridone-5-carboxamide (2-PY)	Overall	107,847	83,673	0.005	50,871	96,757	148,176	208,144	273,875	574,801
N-Methylnicotinamide	Evening	30,778	29,976	0.000	10,503	23,966	38,905	69,048	89,969	171,185
N-Methylnicotinamide	Morning	33,080	29,755	0.030	12,923	26,846	44,939	64,763	71,618	204,001
N-Methylnicotinamide	Overall	31,936	29,842	0.000	11,632	25,061	42,920	67,043	83,576	204,001
N,N-dimethylglycine	Evening	17,988	13,539	0.117	8,203	14,062	25,016	36,204	38,871	95,529
N,N-dimethylglycine	Morning	18,510	12,083	0.069	9,669	15,763	25,517	35,843	40,913	61,383
N,N-dimethylglycine	Overall	18,251	12,811	0.069	9,282	14,756	25,130	35,997	39,493	95,529
Pseudourine	Evening	274,598	151,765	15,726	166,399	239,334	358,391	449,380	594,033	790,730
Pseudourine	Morning	294,705	164,493	65,599	188,340	255,059	359,631	504,821	645,058	1,026,872
Pseudourine	Overall	284,712	158,377	15,726	171,385	251,796	359,338	481,530	625,711	1,026,872
Pyruvate	Evening	324,606	212,715	31,733	157,121	269,787	463,262	573,581	691,224	1,246,452
Pyruvate	Morning	358,240	276,642	33,830	148,280	296,919	484,997	638,380	758,993	1,936,502
Pyruvate	Overall	341,525	247,151	31,733	154,616	278,476	475,254	604,375	744,317	1,936,502
Succinate	Evening	116,003	104,084	14,620	62,727	98,253	141,860	202,105	237,359	1,178,282
Succinate	Morning	114,543	70,694	19,879	64,170	99,112	146,900	208,820	248,076	501,072
Succinate	Overall	115,269	88,739	14,620	63,025	98,866	144,894	207,201	246,081	1,178,282
Taurine	Evening	810,300	1,057,383	42,454	270,940	578,944	887,172	1,575,511	2,366,420	10,369,244
Taurine	Morning	717,446	571,549	62,914	308,395	537,022	1,021,051	1,361,522	1,708,042	4,378,387
Taurine	Overall	763,593	848,500	42,454	279,249	562,006	966,677	1,384,973	1,947,588	10,369,244

Trigonelline	Evening	205,782	197,170	6,702	74,386	129,846	300,630	454,944	546,936	1,411,260
Trigonelline	Morning	197,286	219,104	24,140	71,332	117,324	223,772	467,546	699,587	1,300,831
Trigonelline	Overall	201,508	208,220	6,702	71,438	121,239	255,504	456,633	642,672	1,411,260
Trimethylamine N-oxide	Evening	630,528	1,064,612	52,752	216,408	358,942	578,633	1,158,908	2,082,305	8,903,076
Trimethylamine N-oxide	Morning	531,494	962,756	53,052	213,066	391,264	589,264	899,752	1,050,595	11,991,882
Trimethylamine N-oxide	Overall	580,712	1,014,333	52,752	214,370	366,878	586,225	938,519	1,544,865	11,991,882
Uracil	Evening	256,415	155,592	22,721	128,854	230,558	356,140	469,627	531,072	783,634
Uracil	Morning	290,319	269,211	48,135	143,752	222,749	354,730	502,861	652,762	2,595,744
Uracil	Overall	273,469	220,522	22,721	139,279	227,925	356,161	486,096	585,779	2,595,744
Valine	Evening	84,955	52,388	5,910	46,175	74,025	112,657	150,261	190,080	266,624
Valine	Morning	90,339	56,478	9,124	47,482	77,030	124,296	162,875	190,060	307,094
Valine	Overall	87,663	54,468	5,910	46,343	75,177	121,907	155,891	192,127	307,094
